# DNA hypomethylation characterizes genes encoding tissue-dominant functional proteins in liver and skeletal muscle

**DOI:** 10.1038/s41598-023-46393-5

**Published:** 2023-11-05

**Authors:** Hideki Maehara, Toshiya Kokaji, Atsushi Hatano, Yutaka Suzuki, Masaki Matsumoto, Keiichi I. Nakayama, Riku Egami, Takaho Tsuchiya, Haruka Ozaki, Keigo Morita, Masaki Shirai, Dongzi Li, Akira Terakawa, Saori Uematsu, Ken-ichi Hironaka, Satoshi Ohno, Hiroyuki Kubota, Hiromitsu Araki, Fumihito Miura, Takashi Ito, Shinya Kuroda

**Affiliations:** 1 Department of Biological Sciences, Graduate School of Science, The University of Tokyo, 7-3-1 Hongo, Bunkyo-Ku, Tokyo, 113-0033 Japan; 2 Data Science Center, Nara Institute of Science and Technology, 8916‑5 Takayama, Ikoma, Nara Japan; 3 Department of Omics and Systems Biology, Graduate School of Medical and Dental Sciences, Niigata University, 757 Ichibancho, Asahimachi-Dori, Chuo-Ku, Niigata City, Niigata 951-8510 Japan; 4 Department of Computational Biology and Medical Sciences, Graduate School of Frontier Sciences, The University of Tokyo, 5-1-5 Kashiwanoha, Kashiwa, Chiba 277-8562 Japan; 5 Department of Molecular and Cellular Biology, Medical Institute of Bioregulation, Kyushu University, 3-1-1 Maidashi, Higashi-Ku, Fukuoka, 812-8582 Japan; 6 Bioinformatics Laboratory, Institute of Medicine, University of Tsukuba, Ibaraki, 305‑8575 Japan; 7 Center for Artificial Intelligence Research, University of Tsukuba, Ibaraki, 305‑8577 Japan; 8 Molecular Genetics Research Laboratory, Graduate School of Science, University of Tokyo, 7‑3‑1 Hongo, Bunkyo‑ku, Tokyo, 113‑0033 Japan; 9 Department of AI Systems Medicine, M&D Data Science Center, Tokyo Medical and Dental University, Tokyo, 113-8510 Japan; 10 Division of Integrated Omics, Medical Research Center for High Depth Omics, Medical Institute of Bioregulation, Kyushu University, 3-1-1 Maidashi, Higashi-Ku, Fukuoka, Fukuoka 812-8582 Japan; 11 Department of Biochemistry, Kyushu University Graduate School of Medical Sciences, Fukuoka, 812-8582 Japan

**Keywords:** Proteome informatics, Epigenomics

## Abstract

Each tissue has a dominant set of functional proteins required to mediate tissue-specific functions. Epigenetic modifications, transcription, and translational efficiency control tissue-dominant protein production. However, the coordination of these regulatory mechanisms to achieve such tissue-specific protein production remains unclear. Here, we analyzed the DNA methylome, transcriptome, and proteome in mouse liver and skeletal muscle. We found that DNA hypomethylation at promoter regions is globally associated with liver-dominant or skeletal muscle-dominant functional protein production within each tissue, as well as with genes encoding proteins involved in ubiquitous functions in both tissues. Thus, genes encoding liver-dominant proteins, such as those involved in glycolysis or gluconeogenesis, the urea cycle, complement and coagulation systems, enzymes of tryptophan metabolism, and cytochrome P450-related metabolism, were hypomethylated in the liver, whereas those encoding-skeletal muscle-dominant proteins, such as those involved in sarcomere organization, were hypomethylated in the skeletal muscle. Thus, DNA hypomethylation characterizes genes encoding tissue-dominant functional proteins.

## Introduction

Each tissue is in a different gene expression and protein state necessary for tissue-specific function despite having identical DNA sequences. Epigenetic modifications are a mechanism that enables different expression states^1^. Among epigenetic modifications, DNA methylation, particularly at CpG sites near the transcription start site (TSS) of a gene, represses the expression of that gene^2,3^. Mammalian tissues exhibit specific DNA methylation patterns^4–6^, which correlate with gene expression^7–9^. However, tissue-specific protein expression is regulated not only by DNA hypomethylation but also by other types of regulation such as transcriptional regulation through transcription factor (TF) networks^10^ and posttranscriptional mechanisms of regulation such as protein stability, translation, degradation, aggregation, post-translational modifications, local microenvironments^11^. Detailed understanding of how DNA methylation integrates with other types of regulation to establish tissue-specific proteomes remains unknown.

Systemic metabolism is controlled through multiple tissues in tissue-specific manners. Liver and skeletal muscle are central regulators of systemic metabolism. Both are primary targets of insulin, and they complementarily regulate each other’s metabolism. Therefore, we focused on liver and skeletal muscle and investigated the relationship between the tissue-specific proteome and DNA methylation. Compared to other organs and tissues, including skeletal muscle, liver has various specialized metabolic enzymes that enable the activity of liver-specific pathways, such as the complement and coagulation systems, the urea cycle, cytochrome P450 (CYP)-related metabolism^12^, and tryptophan metabolism^13^. Glucose metabolic pathways are different in liver and skeletal muscle. In liver, enzymes of gluconeogenesis^14^ and GLUT2^15^, a passive transporter of glucose uptake, are abundant; whereas, in muscle, enzymes of glycolysis and the active transporter of glucose uptake, GLUT4, are present^16^. Enzymes involved in pathways that degrade branched-chain amino acids (BCAAs; leucine, isoleucine, and valine) have high activity in skeletal muscle. Proteins of the sarcomere enable contractile activity of skeletal muscles. DNA hypomethylation is associated with liver-specific expression of genes involved in complement and coagulation systems^17^. Global DNA demethylation triggers gene expression necessary for sarcomere formation during development^18^. Such tissue-specific expression occurred primarily in genes with low numbers of CpG sites in promoter region, while ubiquitous expression occurs in genes with high numbers of CpG sites (CpG island) in promoter region^19–21^. However, a proteome-wide analysis of the relationship between genes exhibiting DNA hypomethylation and tissue-specific or ubiquitous proteins is lacking.

We performed whole-genome bisulfite sequencing (WGBS) of hepatocytes isolated from liver and of skeletal muscle (gastrocnemius skeletal muscle) of wild-type mice and measured the DNA methylome. We examined the differences in the DNA methylome, transcriptome, and proteome between liver and skeletal muscle and found that DNA methylation had a primary effect on the tissue-specific distribution of major metabolic enzymes. In liver, genes encoding metabolic enzymes in gluconeogenesis, the urea cycle, ketone body synthesis, CYP, and complement or coagulation systems were associated with liver-dominant DNA hypomethylation. In skeletal muscle, genes encoding BCAA degradation enzymes and proteins of sarcomeres were associated with DNA hypomethylation. In contrast to the tissue-specific proteins, proteins encoded by genes hypomethylated in both tissues, such as ribosome proteins, were differentially expressed due to post-transcriptional differences between liver and muscle.

## Results

### Overview of this study

**Figure 1:**
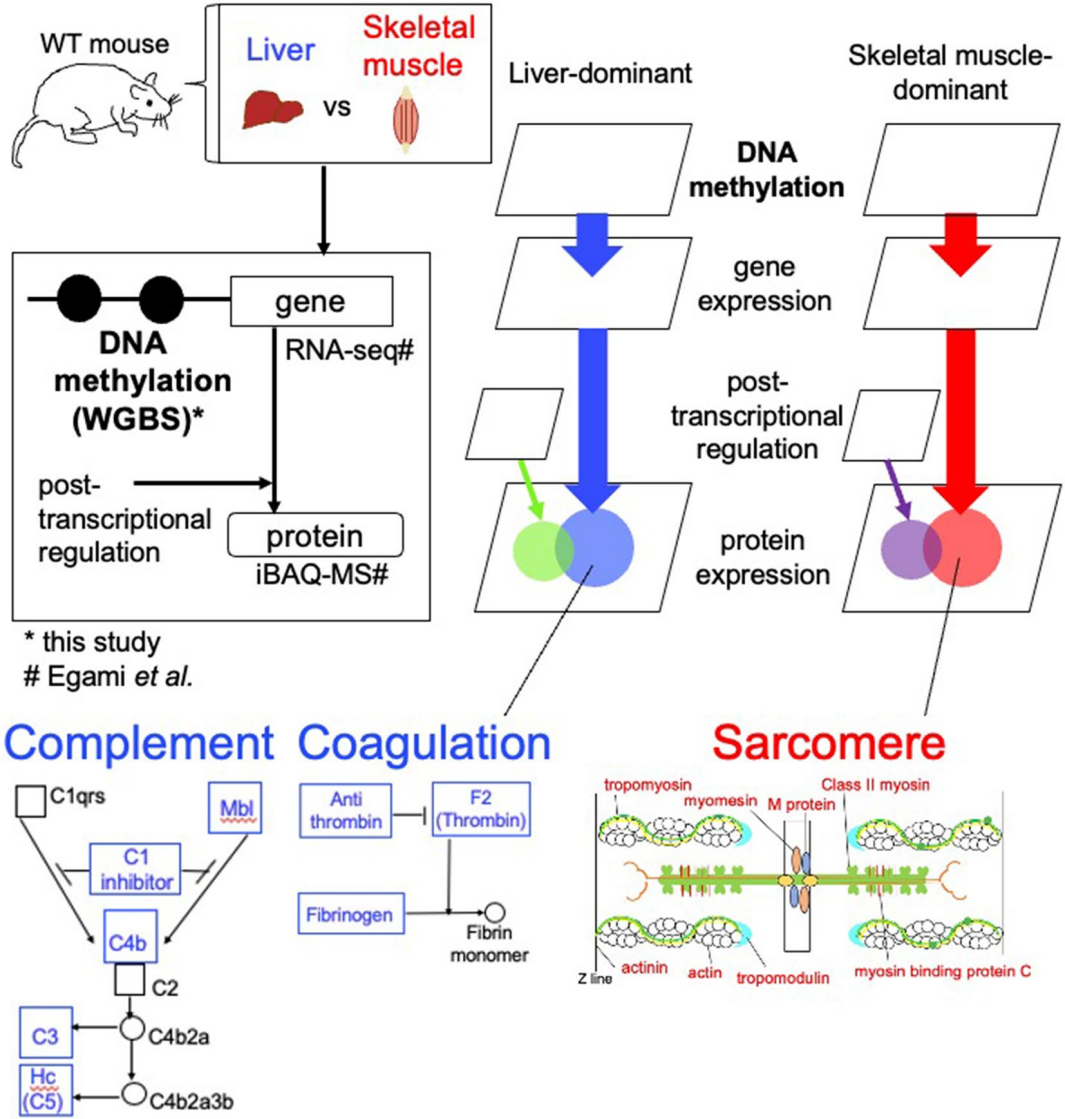
Overview of this study. DNA methylation ratios, gene expression levels, and protein expression levels were obtained from the liver and skeletal muscle of wild-type mice. We compared these omics data between the liver and skeletal muscle, and genes with different DNA methylation statuses, genes with different expression levels, proteins with different post-transcriptional states, and proteins with different expression levels were identified. Using these results, we explained liver- skeletal muscle-dominant functional protein expression, providing an example of a pathway where proteins with DNA hypomethylation are abundant.

In this study, we measured the DNA methylome from mouse liver and skeletal muscle, integrated the data with the transcriptome and proteome of these mouse tissues^22,23^, and examined how tissue-dominant protein and gene expression were associated with DNA hypomethylation (Fig. 1). In this study, we measured DNA methylation by WGBS using isolated hepatocytes and gastrocnemius muscle from C57BL6 mice. We used isolated hepatocytes for the DNA methylome measurement and integrated these data with transcriptome and proteome data for entire isolated liver^22,23^. The DNA methylome from hepatocytes in our study was highly similar to that reported for liver of B6Ncrl mice^24^ (*r* = 0.960, Supplementary Fig. 1a), indicating that the DNA methylome of hepatocytes is comparable to that of liver. Therefore, we refer to the hepatocyte DNA methylome as the liver DNA methylome subsequently.

### Association of DNA hypomethylation in promoter and first exon regions with gene expression

**Figure 2:**
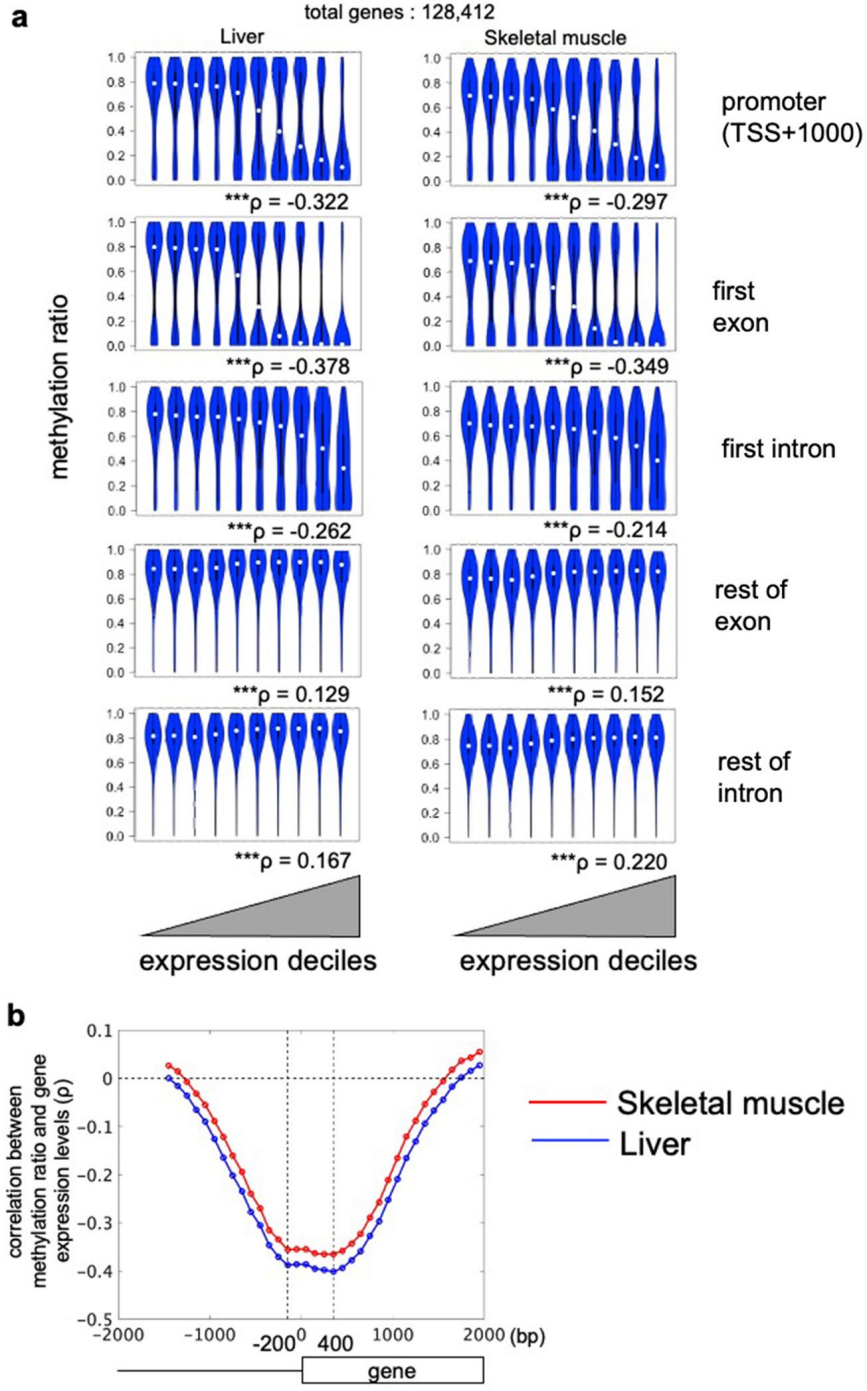
Relationship between gene expression and methylation across gene regions. Each gene is an Ensemble transcript ID unit. Transcripts represented by different IDs but with the same TSS were combined into one. (**a**) Plots show gene expression divided into deciles from low to high and methylation ratios of CpG sites for each of the indicated regions of genes. Correlations between expression and methylation ratio are presented as Spearman's rank correlation coefficient *ρ*. Statistical significance was determined by t-test for correlation coefficient ***p < 0.001). (**b**) Plot shows the Spearman's rank correlation coefficient between the methylation ratios and gene expression in 100-bp regions from 1500 bp upstream to 2000 bp downstream of the TSS. Vertical dotted lines indicate the region of strongest negative correlation (200 bp upstream ~ 400 bp downstream of the TSS). Using a *p* < 0.001 in t-test for correlation coefficient, the region with significant negative correlation was 1200 bp upstream ~ 1500 bp downstream.

We examined the relationship between DNA methylation and gene expression levels for each genomic region to determine regions for which DNA methylation status is associated with gene expression (Fig. 2). We divided the genes into ten sets evenly based on their expression levels (expression decile^7^) (Fig. 2a). For each decile, we examined the distribution of the methylation ratio of CpG sites in each region. We found that the strongest negative correlations between DNA methylation ratios and gene expression levels were in the promoter region (liver: *ρ* = − 0.322***, skeletal muscle: *ρ* = − 0.298***) and the first exon (liver: *ρ* = − 0.378***, skeletal muscle: *ρ* = − 0.349***), indicating that high gene expression is associated with DNA hypomethylation of CpG sites in the region near the TSS in both tissues. These results are consistent with previous studies showing DNA hypomethylation at genes with high expression^8,25,26^.

Because we found a negative correlation between methylation near the TSS and gene expression, we examined the correlation between methylation and gene expression of 100-bp regions near the TSS (Fig. 2b). We found a strong negative correlation of DNA methylation ratio between 200 bp upstream and 400 bp downstream of the TSS and gene expression, and the correlation weakened monotonically outside this region. We used DNA methylation from 200 bp upstream to 400 bp downstream of the TSS for further study.

Note that the non-CpG (CHG and CHH) cytosines were hypomethylated in all regions and were not negatively correlated with expression levels (Supplementary Fig. 1b). Therefore, only methylation of CpGs was included in the analysis in the following sections.

### Identification of differentially hypomethylated genes (DMGs), differentially expressed genes (DEGs), differentially expressed proteins (DEPs), and different protein/mRNA ratio proteins (DRPs)

**Figure 3:**
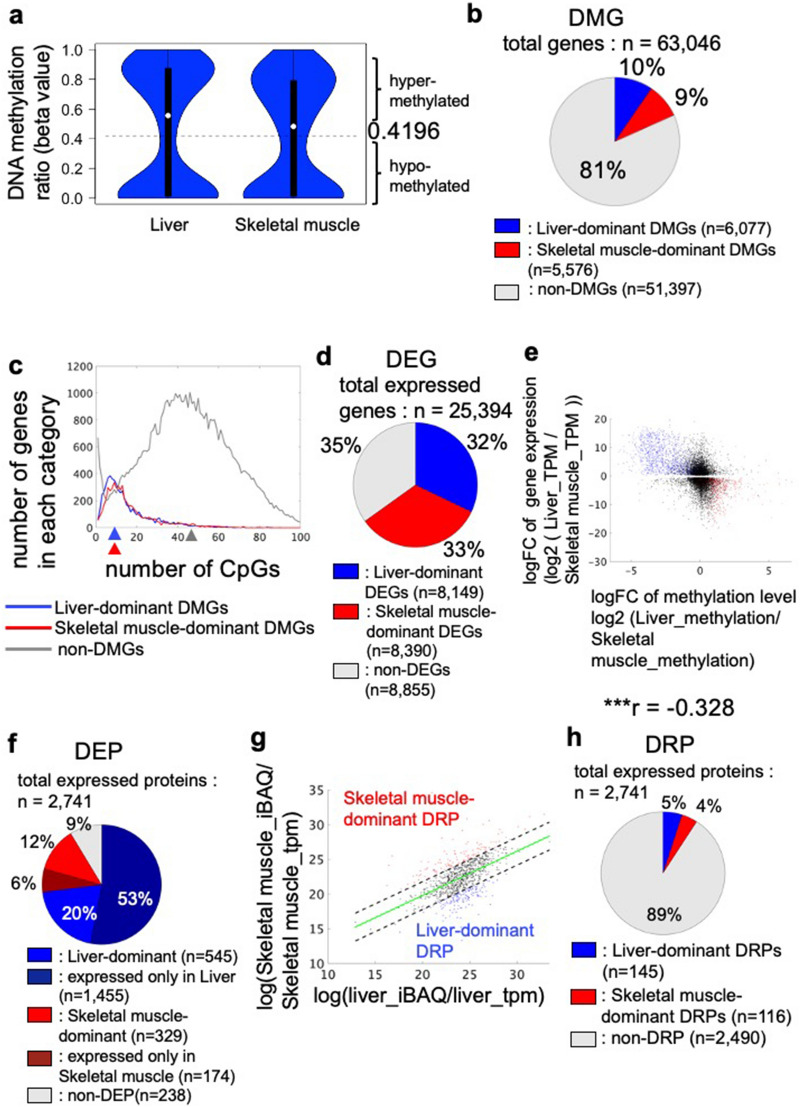
The number and characteristics of DMGs, DEGs, DEPs, and DRPs. (**a**) Distribution of methylation ratios at 200 bp upstream ~ 400 bp downstream of TSS for all genes in liver and in skeletal muscle. By Silverman test [*p* < 0.001 for unimodality and *p* = 0.15 for bimodality], the distribution is bimodal. Dotted line indicates the threshold value (0.4196, determined by Otsu’s method) used to separate the genes with DNA hypermethylation from genes with DNA hypomethylation. (**b**) Pie chart showing the proportion of differentially hypomethylated genes (DMGs) in liver or skeletal muscle. (**c**) The number of liver-dominant or skeletal muscle-dominant DMGs and non-DMGs per CpG number at 200 bp upstream ~ 400 bp downstream of the gene TSS. Median CpG numbers for each group are indicated by colored triangles. (**d**) Pie chart showing the proportion of differentially expressed genes (DEGs) in liver or skeletal muscle. (**e**) Plot shows the relationship between the difference in gene expression [log2 (TPM in liver/TPM in skeletal muscle)] for each DEG and the difference in methylation ratios between liver and skeletal muscle [log2 (methylation ratio in liver/methylation ratio in skeletal muscle)]. Blue dots indicate liver-dominant DEGs with DNA hypomethylation (DMDEGs); red dots indicate skeletal muscle-dominant DMDEGs. Correlation coefficient *r* = − 0.328 and ***p < 0.001 in t-test for correlation coefficient. (**f**) Pie chart showing the proportion of differentially expressed proteins (DEPs) in liver or skeletal muscle. DEPs include those with increased expression in one tissue compared to the other (dominant) and those that were detected in one tissue or the other (only). (**g**) Identification of proteins with different protein/mRNA ratios between tissues (DRPs). The protein/mRNA ratio in skeletal muscle was plotted against the protein/mRNA ratio of each protein in the liver [log ratio of gene expression (TPM) to protein abundance (iBAQ value)]. Proteins that deviated from the regression line (green) were considered to have a higher protein/mRNA ratio between tissues than the other tissue. Blue dots indicate DRPs in liver; red dots indicate DRPs in skeletal muscle. (**h**) Pie chart showing the proportion of DRPs in liver or skeletal muscle.

We observed a bimodal distribution of methylation ratios at this region for all genes in liver and skeletal muscle (Fig. 3a). We set a threshold of 0.4196 by the Otsu method^27^ to divide genes into hyper- and hypomethylated genes.

As expected from studies of mouse and human germ cells^28,29^, hypomethylated genes significantly overlapped with genes in open chromatin, as defined by assay for transposase accessible chromatin with high-throughput sequencing (ATAC-seq) obtained from the ChIP-atlas^30^ (right-tailed Fisher's exact test with *p* < 0.001, Supplementary Fig. 1c). Conversely, the hypermethylated genes had significantly less overlap with accessible genes (*p* < 0.001 in the left-tailed Fisher's exact test, Supplementary Fig. 1d). These results indicated that hypomethylated regions are open chromatin, consistent with the previous observations.

A study of colon cancer reported that DNA methylation negatively correlates with expression, especially when the methylation ratio changes from low to high^31^. To determine if this relationship exists in healthy tissues, we examined the correlation of three cases: the hypomethylated gene in one tissue and the hypermethylated gene in the other; the hypomethylated gene in both tissues; the hypermethylated gene in both tissues. We found the strongest negative correlation between the hypomethylated gene in one tissue and the hypermethylated gene in the other (Supplementary Fig. 1e,* r* = − 0.587). Correlations were moderate for hypomethylated genes in both tissues (*r* = − 0.256), and correlations were the smallest for hypermethylated genes in both tissues (*r* = − 0.083). Because little correlation was found for hypermethylated genes in both tissues, subsequent comparisons of methylation between tissues analyzed genes that were hypomethylated in at least one of the tissues.

We identified differentially hypomethylated genes (DMGs) between the liver and skeletal muscle as follows (see Methods). We identified CpGs with different methylation ratios between liver and skeletal muscle and defined those as differentially hypomethylated CpGs (DMCpGs). We defined genes with at least one DMCpG between 200 bp upstream and 400 bp downstream of the TSS as DMGs (Fig. 3b). The number of DMGs hypomethylated in liver, defined as the liver-dominant DMGs, was 6077 (10%), and the number in skeletal muscle, defined as the skeletal muscle-dominant DMGs, was 5576 (9%). The other 51,397 (81%) genes were non-DMGs.

To explore the characteristics of DMGs, we measured the number of CpGs present between 200 bp upstream and 400 bp downstream of the TSS (Fig. 3c). In both tissues, most DMGs had a lower number of CpG than were found in non-DMGs: The median number of CpGs in DMGs was 10, whereas the median was 47 in non-DMGs (*p* < 0.001). We further delimited the region 200 bp upstream to 400 bp downstream of the TSS into 40 bp sections and determined the number of CpGs in each region for each gene (CpG density vector). We classified these CpG density vectors into two clusters using hierarchical clustering (Supplementary Fig. 1f, Euclidean distance, Ward method). All genes were classified as either high or low CpG density genes. Consistent with the low median number of CpGs in the 600 bp region surrounding the TSS, 85% of DMGs were in the cluster of low CpG density genes (Supplementary Fig. 1f, right). Using data for the mouse from the UCSC Genome Browser^32^, we found that only about 2% of genes with CpG islands were DMGs (Supplementary Fig. 1g). This result is consistent with the previous observation that CpG islands are mostly hypomethylated in mouse liver^33^. Previous studies found that tissue-specific genes have low CpG densities and housekeeping genes have high CpG densities^19–21^, and our results are consistent with these studies.

We examined whether tissue-dominant DMGs associate with the tissue-dominant gene and protein expression on an omics-wide scale. We identified genes differentially expressed in either liver or skeletal muscle [differentially expressed genes (DEGs)] (see “Methods”). Among 25,394 genes expressed in at least liver or skeletal muscle in total, there were 8,149 (32%) liver-dominant DEGs and 8,390 (33%) skeletal muscle-dominant DEGs (Fig. 3d). The remaining 8,855 genes (35%) were non-DEGs.

We found a negative correlation (*r* = − 0.328) between log_2_FC of methylation ratios between the tissues and log_2_FC of gene expression levels of DEGs between the tissues (Fig. 3e), indicating that tissue-dominant gene expression for liver and skeletal muscle is associated with DNA hypomethylation.

We identified liver-dominant differentially expressed proteins (DEPs) (Fig. 3f) as proteins that had a significantly greater abundance (q < 0.01) in liver than skeletal muscle or as proteins present only in liver (see “Methods”). Muscle-dominant DEPs were identified in a similar manner. Among 2741 proteins detected in at least in one of the tissues, 545 (20%) proteins were more abundant in liver than skeletal muscle and 329 (12%) proteins were more abundant in skeletal muscle than liver. Liver had a much higher number [1455 (53%)] of proteins that were unique compared with skeletal muscle that had 174 (6%). Proteins that are more abundance in liver or present only in liver were defined as liver-dominant DEPs. The muscle-dominant DEP was defined in the analogous manner. Proteins that were not DEPs were defined as non-DEPs. Most of proteins were the DEPs (2503 proteins, 91%); only 228 (9%) proteins were non-DEPs.

In addition to the differences in gene expression related to DNA hypomethylation, another possible contributor to protein abundance or presence can be differences in post-transcriptional regulation, including the efficiency of protein translation and differences in protein stability or protein degradation. We considered the protein/mRNA ratio as an indicator of post-transcriptional regulation and we defined proteins with a higher ratio in liver or skeletal muscle as differential protein-per-mRNA ratio proteins (DRPs) (see “Methods”) (Fig. 3g). We identified 145 liver-dominant DRPs (5% of detected proteins) and 116 skeletal muscle-dominant DRPs (4%) (Fig. 3h).

### Association between DMGs, DEGs, DEPs, and DRPs

**Figure 4:**
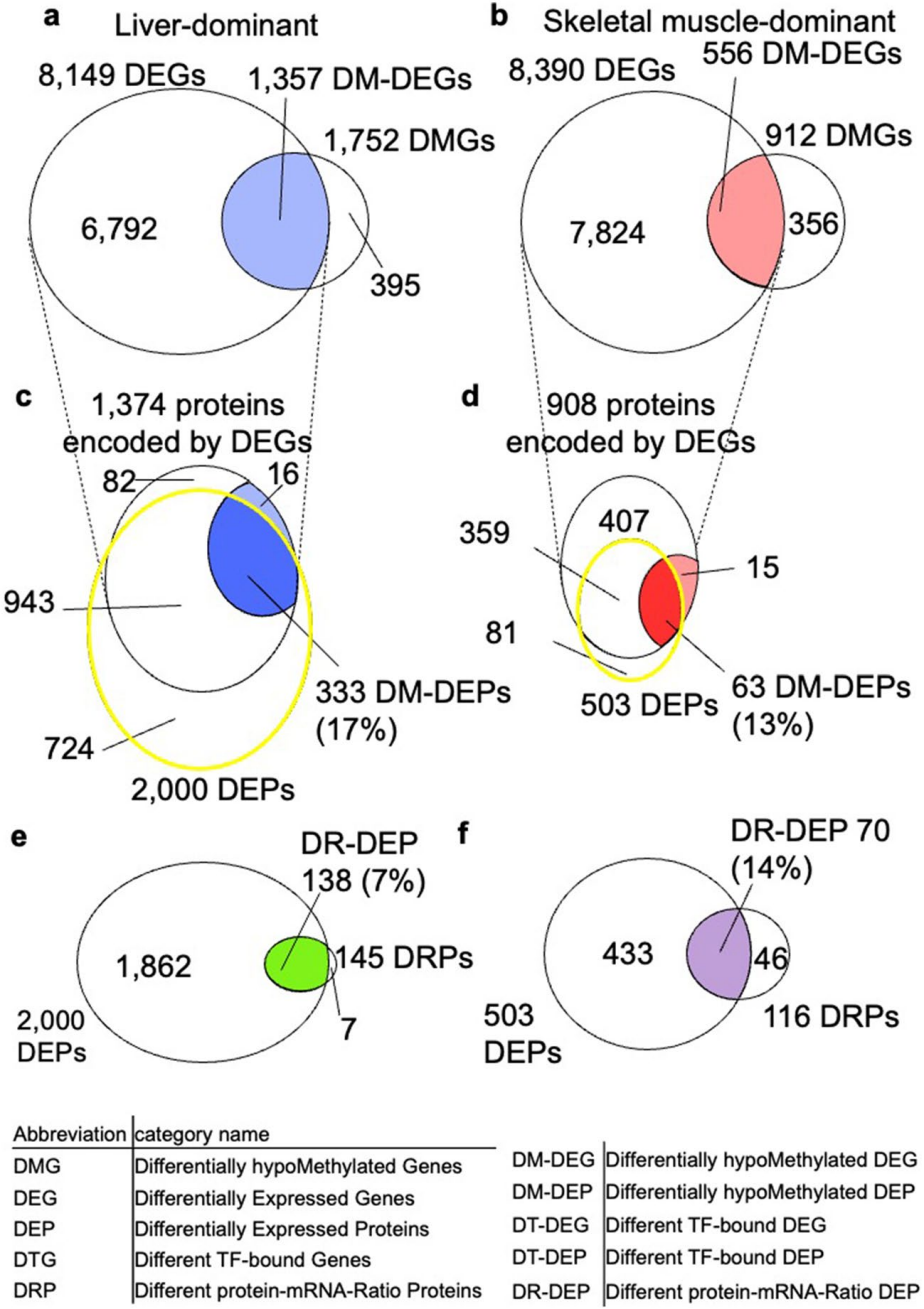
Overlap of DMGs, DEGs, DRPs, and DEPs. (**a**, **b**) Venn diagrams show the relationship among liver-dominant DMGs, DEGs (**a**) or skeletal muscle-dominant DMGs, DEGs, and DEPs (**b**) and how these were used to define DEGs with DNA hypomethylation (DM-DEGs) (**c**, **d**) DEPs produced from DM-DEGs (DM-DEPs). The areas enclosed by the yellow circles are the DEPs in each tissue. The percentage of DM-DEPs in the DEPs for each tissue is indicated. The overlap between liver-dominant or skeletal muscle-dominant DMGs and DEGs was significant at *p* < 0.001 by right-tailed Fisher’s exact test. (**e**–**f**) Venn diagrams of the liver-dominant DEPs and DRPs (**e**) or skeletal muscle-dominant DEPs and DRPs (**f**). DEPs that were also DRPs were defined as DR-DEPs. The percentage of DR-DEPs in among DEPs is indicated.

We analyzed the overlap among DMGs, DEGs, and DEPs (Fig. 4). Among 8149 liver-dominant DEGs and 1752 liver-dominant DMGs, 1353 genes (17% of DEGs) overlapped (Fig. 4a). Among 8390 skeletal muscle-dominant DEGs and 912 skeletal muscle-dominant DMGs, 556 genes (7% of DEGs) overlapped (Fig. 4b). Conversely, there was significantly less overlap between liver-dominant DEGs and skeletal muscle-dominant DMGs (1.5% of DEGs) and between skeletal muscle-dominant DEGs and liver-dominant DMGs (2.3% of DEGs) compared with the overlap within each tissue (*p* < 0.001 for both in left-tailed Fisher’s exact test, Supplementary Fig. 1h,i), indicating that the DEGs are associated with DNA hypomethylation rather than hypermethylation in both liver and skeletal muscle. DEGs that overlapped with DMGs are hereafter referred to as differentially hypomethylated DEGs (DM-DEGs).

We next examined the overlap between DEGs and DEPs. Among 1374 proteins encoded by liver-dominant DEGs, 1276 were liver-dominant DEPs (Fig. 4a, Supplementary Fig. 1j). Among 908 proteins encoded by skeletal muscle-dominant DEGs, 486 genes were skeletal muscle-dominant DEPs (Fig. 4b, Supplementary Fig. 1k). Conversely, there was significantly less overlap between skeletal muscle-dominant DEGs and liver-dominant DEPs and between liver-dominant DEGs and skeletal muscle-dominant DEPs (Supplementary Fig. 1j,k), indicating that the DEGs are associated with DEPs.

We evaluated the overlap between the DM-DEGs and DEPs. In liver, 333 DEPs (17% of the total liver-dominant DEPs) overlapped with DM-DEGs (Fig. 4c); in skeletal muscle, 63 DEPs (13% of the total skeletal muscle-dominant DEPs) overlapped with DM-DEGs (Fig. 4d). DEPs encoded by these DM-DEGs are henceforth referred to as differentially hypomethylated DEPs (DM-DEPs). In both tissues, about 15% of the DEPs were DM-DEPs. We also evaluated the overlap between non-DMGs, non-DEGs, and non-DEPs (Supplementary Fig. 1l,m). 6,590 out of non-DMGs overlapped with non-DEGs (Supplementary Fig. 1l). 79 out of the 238 non-DEPs were encoded by genes that are non-DMGs and non-DEGs (Supplementary Fig. 1m).

We also examined the overlap between DEPs and DRPs (Fig. 4e,f). In liver, 138 of the 145 DRPs overlapped with DEPs (Fig. 4e); in skeletal muscle, 70 of the 116 DRPs overlapped with DEPs. This subset of DEPs is hereafter referred to as DR-DEPs (Fig. 4f).

To assess the contribution of TFs to differences in gene expression, we defined differentially TF-bound genes (DTGs) as those with a TF-binding peak by ChIP-atlas^30^ that differed between liver and skeletal muscle (Supplementary Fig. 2, see “Methods”). Among the TF-bound DTGs in the liver-dominant DMGs, 83% were bound to Foxa1, Ctcf, or Cebpb, or some combination thereof (Supplementary Fig. 2a, see Supplementary text). In contrast, only 34% of TF-bound DTGs in skeletal muscle-dominant DMGs were bound to Myod1, Ctcf, or Cebpb, or some combination (Supplementary Fig. 2b). Based on the overlaps between DTGs, DEGs, and DEPs, we identified DT-DEPs (Supplementary Fig. 3a–d, see Supplementary text).

We performed enrichment analyses of the DM-DEPs, DT-DEPs, and DR-DEPs (Supplementary Fig. 3e,f). The skeletal muscle-dominant DM-DEPs were not enriched in any pathways due to their small number. However, in liver, both DM-DEPs and DT-DEPs were enriched in several pathways belonging to “Metabolism” or “Organismal Systems” and in peroxisome in the “Cellular Process” category. In skeletal muscle, only DT-DEPs exhibited significant enrichment and all pathways were associated with muscle function: calcium signaling pathway, cardiac muscle contraction, and adrenergic signaling in cardiomyocytes (Supplementary Fig. 3f, left). Enrichment analysis identified “Ribosome” as enriched in the liver-dominant DR-DEPs (Supplementary Fig. 3e, left). Because the liver-dominant DR-DEPs largely overlapped with proteins encoded by non-DMGs and non-DEGs (62 in 138 liver-dominant DRPs, Supplementary Fig. 3g), differences in the abundance or presence of ribosome-associated proteins were suggested to be associated with post-transcriptional regulation rather than regulation at the level of gene expression.

One mechanism that results in a discrepancy between protein and mRNA abundance is the regulation of translation through the 5′-terminal oligopyrimidine (5′-TOP) motif, which is mainly present in the mRNAs of ribosomal component proteins^34^. Translation of mRNA with this motif is repressed during starvation^34^. Among the 72 proteins that we detected and that are translated from mRNAs with 5′-TOP motifs, no DR-DEPs with high protein/mRNA ratios in skeletal muscle and 25 were DR-DEPs with high protein/mRNA ratios in liver (Supplementary Fig. 3h,i).

We analyzed TF binding to genes encoding DT-DEPs and the pathways in which the TF-bound genes were associated (see “Methods”). In skeletal muscle, only 3 pathways and one TF showed any significant association: Brd4 was significantly associated with DT-DEPs in oxidative phosphorylation, thermogenesis, and retrograde endocannabinoid signaling (Supplementary Fig. 3f, right). In liver, the pattern was more complex with Cebpb significantly associated with DT-DEPs in 5 pathways, spanning multiple categories, Hdac3 and Nr3c1 associated with complement and coagulation in the Organismal Systems category, and Nr3c1 with glycerophospholipid in the Metabolism category. Peroxisome in the Cellular Process category was significantly associated with Cebpb, Clock, and Ctcf (Supplementary Fig. 3f, right). Cebpb and Brd4 were suggested to be major TFs specific for the liver- and skeletal muscle-dominant DT-DEPs, respectively.

Collectively, these results revealed tissue-specific regulation of functional protein expression by DNA hypomethylation as indicated by the DM-DEPs, post-transcriptional regulation as indicated by the DR-DEPs, and TF-binding status as indicated by the DT-DEPs. In particular, liver showed a more complex regulatory pattern involving more pathways than skeletal muscle, consistent with the diverse functions of liver. Additionally, the pathways enriched in DM-DEPs overlapped with those associated with Cebpb-bound DT-DEPs, suggesting that these two regulatory mechanisms contribute to these pathways. In contrast, DT-DEPs bound by Brd4 were enriched in different pathways than the pathways enriched across all DT-DEPs in skeletal muscle, suggesting that Brd4 and DNA hypomethylation regulated distinct tissue-specific functions.

Possible factors causing DEG and DEP that could not be explained by the analysis so far include epigenomes other than DNA methylation. Therefore, we attempted to explain the DEGs that could not be explained by DMG by histone modifications (HMs) that tend not to coexist with DNA methylation H3K4me3^35^ and H3K27ac^36^, which activate gene expression. Peaks of HMs H3K4me3 and H3K27ac were obtained in liver and peaks in skeletal muscle were obtained from previous studies (H3K4me3 Liver: GSM874957^37^, GSM594589^38^; H3K4me3 Skeletal muscle: GSM2794091^39^, GSM4231198^40^; H3K27ac Liver: GSM2136890^41^, GSM1479723^42^; H3K27ac Skeletal muscle: GSM6475251^43^, GSM2219812^44^; data from the processed peak regions were downloaded from ChIP-atlas^30^). We then identified genes with peaks of HM at 1000 bp before and after the TSS and confirmed that the genes with peaks were consistent across many papers. We identified genes with peaked HMs in liver only and in muscle only (Supplementary Fig. 3j,k). These genes with different HM status between liver and muscle included 1495 of the liver-dominant DEGs that were not overlapped with DMG and 1039 of the skeletal muscle-dominant DEGs (Supplementary Fig. 3l,m). Some genes differed in both DNA methylation and HM, with 587 of the liver-dominant DM-DEGs and 115 of the skeletal muscle-dominant DM-DEGs also differing in HM statuses. Therefore, at least on the numbers, these histone modifications are more overlap with DEGs that are non-DMGs than DM-DEGs.

### Tissue-dominant DNA hypomethylation of liver-specific or skeletal muscle-specific functional proteins

**Figure 5:**
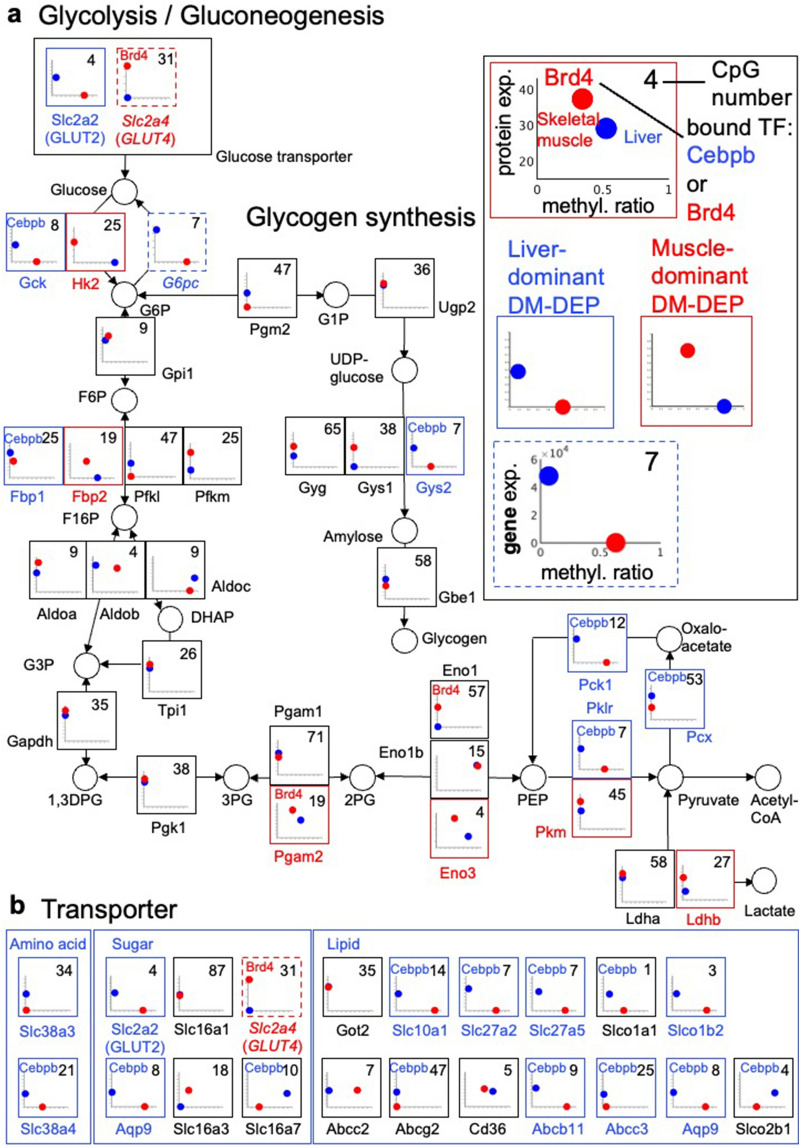
Protein expression or gene expression and methylation ratios of the encoding genes of enzymes in the glucose metabolism and nutrient transporters. (**a**) Glucose transporters and enzymes in the glycolysis/gluconeogenesis and glycogen synthesis pathways. (**b**) Sugar, amino acid, and lipid transporters. Key (“Protein” box): Protein expression (protein exp.) is presented after normalization and ranges from the lowest to the highest expression level for the measured protein. Proteins that were not detected in one tissue are plotted as the value representing the lowest expression level of any detected protein in that tissue. The methylation ratio (methyl. ratio) is the mean methylation ratios of CpGs at 200 bp upstream ~ 400 bp downstream of the TSS. The number in the upper right corner of the plot represents the number of CpGs in this region. Blue circles indicate the value for liver; red circles indicate the value for skeletal muscle. Proteins that were highly expressed and had genes with DNA hypomethylation (DM-DEPs) in one tissue typically appear as dots in the upper left and lower right of the graph. Blue letters in the protein name and blue boxes indicate liver-dominant DM-DEPs. Red letters and red boxes indicate skeletal muscle-dominant DM-DEPs. DEGs for which we did not have corresponding protein measurements are presented as plots of gene expression (gene exp. in TPM) against DNA methylation ratio (methyl. ratio) and the plots are outlined with dashed boxes (see *G6pc* and *GLUT4* as examples). Proteins marked with “Cebpb” on the plot are DT-DEPs bound by Cebpb (see Gck as an example) and proteins marked by “Brd4” are DT-DEPs bound by Brd4 (see Pgam2 as an example). Supplementary Table 3 provides the full name of the enzymes or proteins.

**Figure 6:**
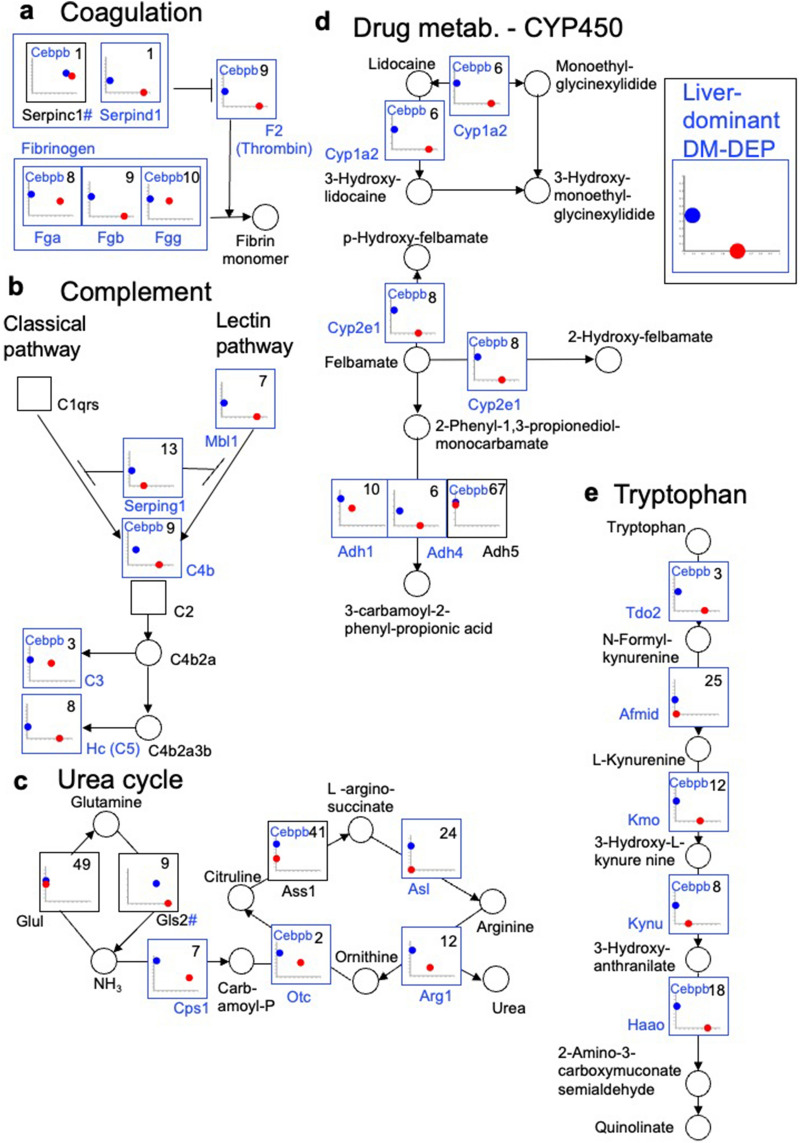
Protein expression levels and methylation ratios of enzymes in functional or metabolic pathways in the liver. (**a**) coagulation system, (**b**) complement system (classical and lectin pathways), (**c**) urea cycle, (**d**) CYP450-related drug metabolism, (**e**) tryptophan metabolism. ^#^represents proteins encoded by highly methylated genes in both liver and muscle but differ significantly between the liver and skeletal muscle in both methylation and protein expression. See Fig. 5 for detailed key to plots of expression levels versus methylation ratios for encoding genes. A representative pattern for liver-dominant DM**-**DEPs is provided.

**Figure 7:**
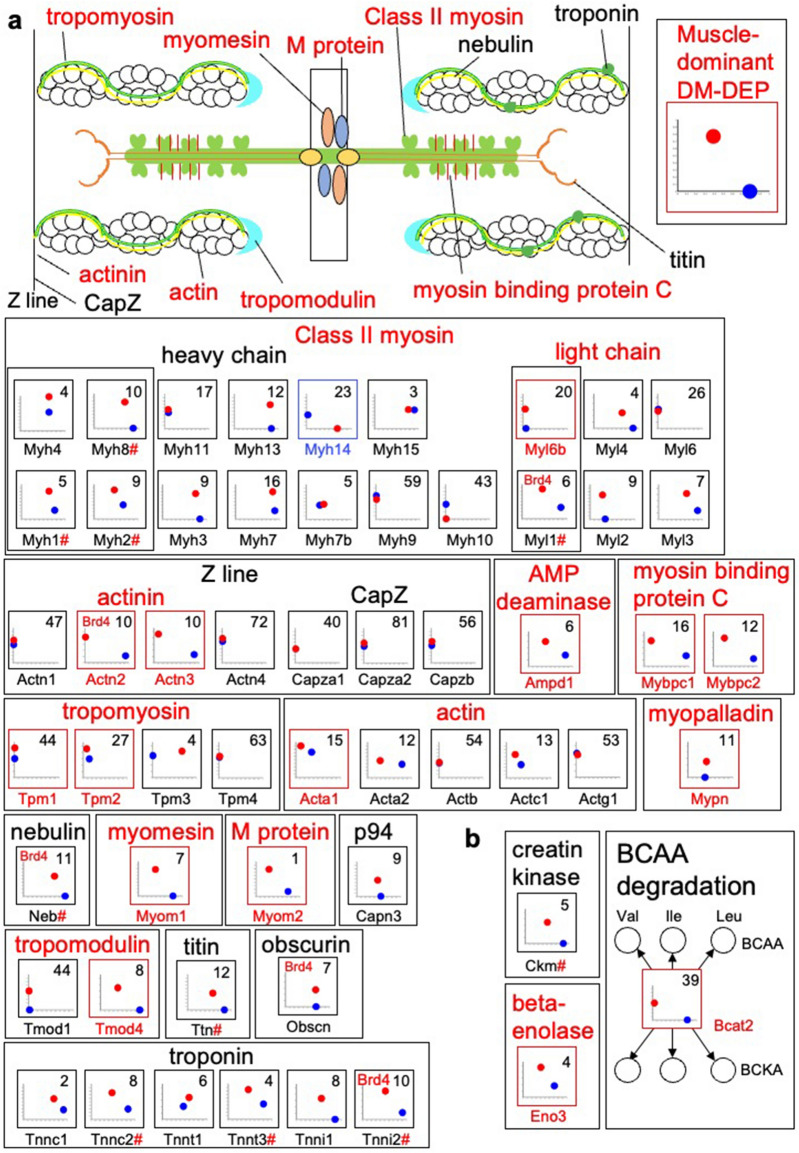
Expression levels and methylation ratios of sarcomere component proteins and enzymes of BCAA degradation. (**a**) Component proteins of the sarcomere, including enzymes involved in providing energy for contraction, are presented with a diagram of the sarcomere. (**b**) Enzymes of BCAA degradation. Bcat1 was not detected at the protein or transcript level. BCKA: branched-chain keto acid. ^#^represents proteins that are hypermethylated in both liver and muscle but differ significantly between the liver and skeletal muscle in both methylation and protein expression. See Fig. 5 for detailed key to plots of expression levels versus methylation ratios for encoding genes. A representative pattern for skeletal muscle-dominant DM**-**DEPs is provided.

We examined the DNA methylation status and protein expression status for primary functions in liver and skeletal muscle at the level of individual genes and proteins (Figs. 5, 6, 7, and Supplementary Fig. 4). Both liver and skeletal muscle utilize glucose for cellular metabolism, however, each has tissue-specific isoforms or orthologs along the pathways.

We examined the DNA methylation of genes encoding enzymes in glycolysis/gluconeogenesis along with the abundance of the protein, or if protein abundance was not measured in the proteomic data, the amount of mRNA in the transcriptomic data (Fig. 5a). Because we found Cebpb was associated with liver-dominant DM-DEPs (Supplementary Fig. 3e) and Brd4 was associated with skeletal muscle dominant DM-DEPs (Supplementary Fig. 3f), we also evaluated if these TFs were bound to the genes in the ChIP-atlas data.

For glycolysis, the rate-limiting enzymes responsible for converting glucose to G6p are also specific in liver and skeletal muscle: Gck is the liver-specific enzyme and Hk2 is the skeletal muscle-specific enzyme^45,46^. As expected, Gck was a liver-dominant DM-DEP and Hk2 was a skeletal muscle-dominant DM-DEP (Fig. 5a). However, only Gck was encoded by a gene bound to Cebpb. The liver-specific and skeletal muscle-specific pyruvate kinases, Pklr and Pkm^47^, were also liver-dominant and skeletal muscle-specific DM-DEPs, respectively.

Gluconeogenesis occurs in liver, not in skeletal muscle. G6pc and Pck1 are rate-limiting enzymes that catalyze irreversible reactions in gluconeogenesis^14^. *G6pc* was a DM-DEG and Pck1 was a Cebpb-associated DM-DEP (Fig. 5a). The protein, G6pc, could not be quantified because unique peptides for mouse G6pc were not identified in the Ensembl database. Another rate-limiting enzyme in glucose metabolism is catalyzed by tissue-specific enzymes. Fbp1 is the liver-specific isoform^48^ and was a liver-dominant DM-DEP that was associated with Cebpb, and Fbp2, skeletal muscle-specific isoform^48^, was a skeletal muscle-dominant DM-DEP (Fig. 5a). Glucose-alanine pyruvate carboxylase Pcx, which is essential for the glucose-alanine cycle in liver, was a liver-dominant DM-DEP that was associated with Cebpb. The three skeletal muscle enzymes Pgam2^49^, Eno3^50^, and Ldhb^51^ were skeletal muscle-dominant DM-DEPs and Brd4 was associated with Pgam2. In contrast, some of the common glycolytic enzymes expressed in both tissues, such as Aldoa, Gapdh, Pgk1, and Tpi, were hypomethylated in both tissues. Many of the liver-dominant or skeletal muscle-dominant rate-limiting enzymes of glycolysis and gluconeogenesis were encoded by genes with DNA hypomethylation, suggesting that the tissue-specific expression of rate-limiting metabolic enzymes is associated with DNA hypomethylation.

Both liver and skeletal muscle synthesize and break down glycogen. For glycogen synthesis, Gys2 was the only liver-dominant DM-DEP. Other enzymes were encoded by hypomethylated genes in both liver and skeletal muscle (Fig. 5a).

Among the glucose transporters, GLUT2 is the liver-specific form^15^. Consistently, we found that GLUT2 protein was present and the encoding gene (*Slc2a2*) was hypomethylated only in liver (Fig. 5a,b). In contrast, skeletal muscle-specific glucose transporter GLUT4^16^ was a skeletal muscle-dominant DM-DEG, although the protein was not measured in this study because of insolubility of GLUT4 (Fig. 5a,b). In addition to the glucose transporters GLUT2 and GLUT4, the sugar transporter encoded by *Aqp9* was a liver-dominant DM-DEP (Fig. 5b). Of the 13 lipid transporters, 7 lipid transporters were liver-dominant DM-DEPs. and 2 amino acid transporters (Slc38a3 and Slc38a4) were DM-DEPs (Fig. 5b). Except for GLUT2 and Slc38a3, all of the other liver-dominant transporters were encoded by genes associated with both DNA hypomethylation and Cebpb. Cebpb was also associated with 4 transporters present in both tissues: Slco1a1, Slco2b1, Slc16a7, and Abcg2.

The liver has specific functions in the complement and coagulation systems, urea cycle, drug metabolism, and tryptophan metabolism (Fig. 6). For both complement and coagulation systems, almost all the DEPs were DM-DEPs (Fig. 6a,b). Among these DM-DEPs, the genes encoding Fgb, Fgg, F2, and Serpinc1 are reportedly hypomethylated specifically in mouse liver^17^, which is consistent with our results. For the urea cycle, Otc, Asl, and Arg1 of the liver-specific detoxification pathway^52^ were DM-DEPs (Fig. 6c). Many liver-dominant DM-DEPs were also present in CYP450-related drug detoxification^12^ and most were associated with Cebpb (Fig. 6d). Tryptophan metabolism is another pathway with many liver-dominant DM-DEPs and only Afmid was not associated with Cebpb (Fig. 6e).

A similar pattern of liver-dominant DM-DEPs with many associated with Cebpb was also observed for other liver functions, such as bile acid synthesis, alcohol degradation, ketone synthesis, and estrogen degradation (Supplementary Fig. 4a–d).

Skeletal muscle is a specialized tissue for contraction, and the sarcomere is a molecular functional unit for contraction. We found 12 sarcomeric proteins were DM-DEPs (Fig. 7a): Myl6b, Actn2, Actn3, Tpm1, Tpm2, Myom1, Myom2, Tmod4, Mybpc1, Mybpc2, Acta1, and Mypn. Not all of the proteins in each family are specific to skeletal muscle, therefore we expected to only detect a subset of myosins and actins, including Acta1^53^, Myh1, Myh2, Myh4, Myh8, Myl1, and Myl6b^54^. Several other skeletal muscle-specific DEPs were not DM-DEPs because the encoding genes were hypomethylated in both liver and skeletal muscle. However, several of these skeletal muscle-dominant DEPs, including the myosins Myh1, Myh2, Myh8, and Myl1; troponins Tnnc2, Tnnt3, and Tnni2; nebulin (Neb); and titin (Ttn), were encoded by genes with methylation ratios that were significantly lower in skeletal muscle than in the liver.

We also found skeletal muscle-specific metabolic enzymes were DM-DEPs: beta-enolase 3 (Eno3), which is involved in glycolysis, and Bcat2, which is involved in BCAA degradation^55^ (Fig. 7b). We did not detect Bcat1 in either tissue. Additionally, the creatine kinase subunit M (Ckm), which provides energy to support metabolism during skeletal muscle contraction^56^, was not a DM-DEP but the encoding gene had significantly lower methylation ratios in skeletal muscle than liver. Brd4 was only associated with 3 skeletal muscle-dominant DEPs of the sarcomere, suggesting that other TFs are sarcomere-specific TFs.

We investigated blood proteins produced in and released by liver or skeletal muscle or both^57,58^ (Supplementary Fig. 4e,f). Liver produces general transport protein albumin (Alb) and other thyroid hormone transport proteins, such as transthyretin (Ttr)^59^. Alb and Ttr were not DEPs, likely because they are released from liver into the blood. However, both were encoded by liver-dominant DM-DEGs (Supplementary Fig. 4e). Among hepatokines, only angiopoietin-like 3 (Angptl3) was a liver-dominant DEP (Supplementary Fig. 4f). Several others, including Fetuin-A (Ahsg) and insulin-like growth factor 1 (Igf1)—both of which target skeletal muscle among other tissues—Angtpl8, Hepassocin (Fgl1), and leukocyte cell-derived chemotaxin 2 (Lect2), were encoded by liver-dominant DM-DEGs but the proteins were not detected. Similarly, we did not detect myokines at the protein level, but we found that myostatin (Mstn), interleukin 6 (Il6), and secreted protein acidic and rich in cysteine (Sparc) were encoded by skeletal muscle-dominant DM-DEGs.

### Hypomethylation of genes encoding proteins with ubiquitous functions in liver and skeletal muscle

**Figure 8:**
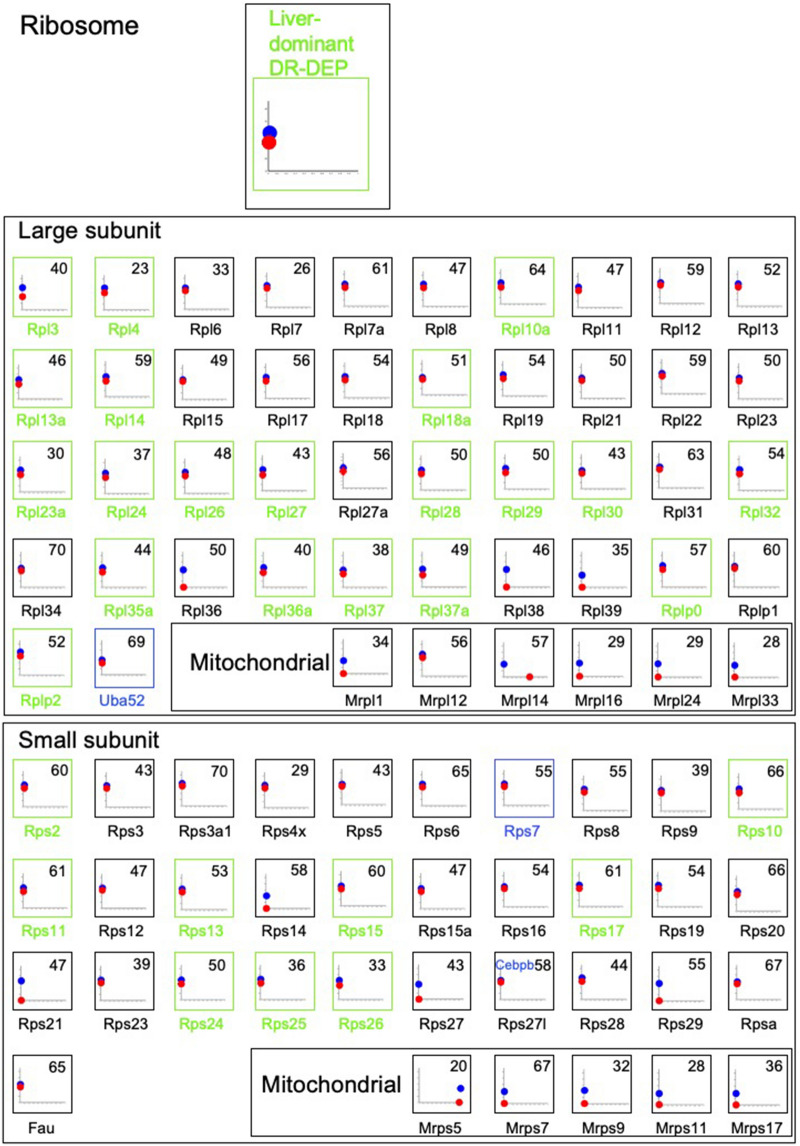
Expression levels and methylation ratios of ribosomal proteins. Green letters in the protein name and green boxes indicate liver-dominant DR-DEPs. See Fig. 5 for detailed key to plots of expression levels versus methylation ratios for encoding genes. A representative pattern for liver-dominant DR-DEPs is provided. Blue letters in the protein name indicate liver-dominant DM-DEGs.

Many ubiquitously expressed proteins of the ribosome (Fig. 8), proteins in the endoplasmic reticulum (ER) and Golgi, as well as the ubiquitin–proteasome pathway (Supplementary Fig. 5) were encoded by hypomethylated genes but these were not DMGs, indicating that these genes are commonly hypomethylated in both tissues. However, enrichment analysis indicated that the liver-dominant DR-DEPs were enriched in “Ribosome” category (Supplementary Fig. 3e), suggesting that the liver-dominant protein expression of these DEPs resulted from post-transcriptional regulation, such as factors affecting translation efficiency or protein stability.

Consistent with the analysis of CpG numbers in all DMGs and non-DMGs across both tissues (Fig. 3c), the CpG numbers of the DMGs encoding proteins in each pathway (median = 19 for glycolysis/gluconeogenesis, 9 for transporters, 8 for complement/coagulation, 12 for sarcomeres, 9 for total liver-dominant DMGs, and 14 for total skeletal muscle-dominant DMGs) were lower than those of the proteins encoded by non-DMGs (median = 47 for glycolysis/gluconeogenesis, 47 for transporters, 47 for sarcomeres, 50 for ribosomes, 55 for protein processing in ER, and 49 for total non-DMGs) (Supplementary Fig. 6).

We also examined the relationship between methylation ratios and gene expression levels instead of proteins expression levels (Supplementary Fig. 7). As in the case of proteins, there were many DM-DEGs in genes encoding enzymes responsible for important tissue functions.

## Discussion

We investigated the differences in protein expression between the liver and skeletal muscle in association with differences in DNA hypomethylation, in TF-binding status, and in post-transcriptional regulation. We measured the methylome in the liver (hepatocyte) and skeletal muscle of wild-type mice by WGBS and integrated those data with transcriptome and proteome data from previous studies^22,23^, data for TF binding obtained from databases. By combining the methylome data with the proteomic and transcriptomic data, we gained insight into epigenetic regulation of tissue-specific protein expression. By integrating the proteomic and transcriptomic data, we gained insight into post-transcriptional regulation using protein/mRNA ratios. Using all this information, we identified differentially methylated genes (DMGs), different TF-bound genes (DTGs), differentially expressed genes (DEGs), different protein/mRNA-ratio proteins (DRPs), and differentially expressed proteins (DEPs) and explored regulatory mechanisms controlling both tissue-specific and ubiquitously expressed proteins. Many tissue-specific functional proteins were associated with DNA hypomethylation and binding to specific TFs. Proteins with ubiquitous functions were encoded by non-DMGs and protein expression was regulated by post-transcriptional mechanisms rather than by gene expression. Considering that DNA methylation is generally considered less variable compared to transcription factor binding and post-transcriptional regulation, it is biologically reasonable to hypothesize that proteins required only in specific organs are regulated by DNA methylation, whereas proteins with potential roles in multiple organs are likely regulated by mechanisms other than DNA methylation such as post-transcriptional mechanisms to achieve tissue dominance.

A comparison of WGBS and transcriptome data from mouse liver and skeletal muscle measured in this study showed a negative correlation between methylation and gene expression levels. This negative correlation was confirmed for methylation in the promoter, first exon, and first intron regions. This result is consistent with previous studies that methylation of the promoter region is negatively correlated with gene expression^2,3^. This result is also consistent with studies using reduced representation bisulfite sequences (RRBS) on European sea bass skeletal muscle and testis^7^, which showed a negative correlation between DNA methylation status near the TSS and gene expression levels. However, our findings differed in that methylation of the first exon had the strongest negative correlation with gene expression levels in our results, whereas the previous study reported a stronger negative correlation between methylation of the first intron and expression levels. Another difference is that methylation of promoters of genes is not significantly correlated with the expression level in skeletal muscle in this previous study^7^. Methodological differences (WGBS versus RRBS) or the analysis of divergent organisms (mammals versus fish) could underlie the different findings. Another previous study using the Sequence Tag Analysis of Methylation Pattern (STAMP) assay for human T cells showed that methylation of the first exon had the highest negative correlation^8^, which is consistent with our results. Several studies^60,61^ also report that hypomethylation of specific promoters is associated with increased expression in mouse liver and skeletal muscle, the organs used in this study.

Because previous studies found that low CpG density corresponds to tissue-specific expression and high CpG density to housekeeping genes and suggested a similar relationship between tissue-specific DNA hypomethylation and CpG density^19–21^, we assessed the relationship between DNA hypomethylation and CpG number. Genes with liver- or skeletal muscle-dominant DNA hypomethylation mainly have lower CpG numbers than genes with DNA hypomethylation in both liver and skeletal muscle. Thus, our results are consistent with the previous studies^19–21^. Furthermore, DNA hypomethylation was associated with genes encoding differentially expressed proteins involved in the major functions of both liver and skeletal muscle. One such major function of the liver, the complement coagulation system, Fgb, Fgg, F2, and Serpinc1, has been reported in previous studies to have high gene expression with liver-specific hypomethylation^17^, and our results, including protein expression, are consistent with this previous study.

Our finding that ribosome-associated genes were hypomethylated in both liver and skeletal muscle and that liver-dominant DRPs were enriched in this pathway indicated that post-transcriptional mechanisms are suggested to control differential production of these proteins. We examined the 5′-TOP motif, a motif involved in translational repression during starvation^34^, as a possible cause of DRPs. The 5′-TOP motif overlapped with liver-dominant DR-DEP and did not overlap with muscle-dominant DR-DEP. These results suggested that starvation represses protein synthesis in skeletal muscle through 5′-TOP-mediated translational repression, whereas this mechanism is less active in liver, leading to higher protein/mRNA in the liver compared to skeletal muscle.

A limitation of this study is that WGBS was performed on hepatocytes, whereas RNA-seq and iBAQ-MS were performed on whole livers. In a previous study, a sufficient correlation of gene expression levels (*r*^2^ = 0.88) was obtained between whole liver and hepatocytes at 11 h post-extraction^62^. Therefore, we expect little difference between the hepatocyte and liver data. Additionally, future studies need to verify with biochemical experiments whether gene hypomethylation results in increased abundance of the DMDEPs identified here. CRISPR/Cas9^63^ can be used to demethylate specific genes in liver or skeletal muscle primary cells or cell lines and both mRNA and protein production can be monitored. If the abundance increases, then these genes are activated by DNA hypomethylation.

Other limitations of this study are that we only linked DNA hypomethylation, binding of TFs, and histone modifications H3K4me3 and H3K27ac near the TSS to gene expression levels. However, methylation and binding of factors in enhancers and insulators also affect gene expression. Therefore, it is necessary to investigate the DNA methylation status and factor binding in these regions in the future and incorporate them into the analysis. Future studies are needed to address the contribution of other histone modifications, to tissue-specific and ubiquitous protein distribution. We simply linked TFs with a binding peak at 1000 bp before and after TSS to DEG and DEP, but more sophisticated methods such as Lisa^64^ may reveal new regulatory networks. Furthermore, transcriptomes and proteomes were linked using the same method as in our previous study^23^, but it is possible that new key molecules can be found by using more sophisticated methods such as DIABLO^65^ of integrating omics. Although we observed tissue differences in mRNA/protein ratios in this study, the specific mechanisms (translation, degradation) have not been identified.

DNA methylation status is regulated by the methyltransferase Dnmt^66,67^, which interacts with other epigenomic factors, and a demethylation-related factor Tet^68^, which is affected by metabolite status and environmental factors^69,70^. We plan to incorporate such upstream factors of DNA methylation into the analysis in future studies.

Here, we explored contribution of DNA hypomethylation to the difference in protein expression between liver and skeletal muscle. The results suggested that DNA hypomethylation explains 15% of the differentially expressed proteins, which include proteins performing key functions in the liver and skeletal muscle. Mouse WGBS data are available in ENCODE^24^, and an atlas of human WGBS data has recently been published^6^. In the future, we plan to extend this study to multiple organs using a such atlas and expand the study to systemically investigate tissue-specific regulation by DNA methylation.

## Methods

### Mouse studies

With the exception of the isolation of primary hepatocytes, all procedures involving animal experiments were approved by the University of Tokyo Animal Ethics Committee. The isolation of primary hepatocytes from mice was approved by the Kyushu University Animal Ethics Committee. All animal experiments were in accordance with the ARRIVE guidelines and the University of Tokyo guidelines for the care and use of laboratory animals. Ten-week-old male C57BL/6J wild-type (WT) mice purchased from SLC Japan, Inc., and acclimated to the laboratory for 0.5 days. Subsequently, after a 16-h fasting period, the mice were euthanized by cervical dislocation between 10:00 and 11:00 AM. Liver samples (whole or left lateral lobes for transcriptome and proteomics,) and skeletal muscle (gastrocnemius) were dissected and immediately frozen in liquid nitrogen. We used n = 3 for the methylome. Transcriptome and proteome acquisition experiments were performed in our previous study^23^. Briefly, frozen liver and skeletal muscle were ground to a fine powder in a blender with dry ice, and used for transcriptome, and proteome analysis, n = 11 for the transcriptome, and n = 5 for the proteome.

### Isolation of primary hepatocytes

Primary hepatocytes were isolated from 10-week-old male C57BL/6J WT mice and cultured using the following method^71^. The liver of anaesthetized mice was perfused at a rate of 4.5 mL/min for the first 2 min with perfusion solution (Hank’s balanced salt solution (Thermo Fisher Scientific, Waltham, MA) containing 10 mM HEPES and adjusted to pH 7.4 with NaOH), and then for 20 min with perfusion solution with collagenase type I (0.3 mg/mL) (Worthington, Lakewood, NY) and complete EDTA-free protease inhibitor cocktail (Roche, Basel, Switzerland). Hepatocytes from C57BL/6J were purified by density gradient centrifugation with Percoll (Sigma-Aldrich, St. Louis, MO). Isolated hepatocytes were seeded at 5.0 × 10^4^ cells/cm^2^ to collagen I-coated dish and cultured with DMEM (Sigma-Aldrich, St. Louis, MO) supplemented with 10% fetal bovine serum (NICHIREI BIOSCIENCES, Japan), Penicillin/Streptomycin (10,000 U/mL) (Thermo Fisher Scientific, Waltham, MA). After 24 h, the medium was replaced with serum-free DMEM containing 0.01 nM insulin and 10 nM dexamethasone (Fujifilm-Wako, Japan) and incubated for 16 h.

### Omics analysis

Genomic DNAs from hepatocytes and skeletal muscle was prepared using DNeasy Blood & Tissue Kit (QIAGEN, Hilden, Germany) according to the manufacturer's instructions. The library preparation for WGBS was performed with the tPBAT protocol described previously^72^. In brief, 100 ng of purified genomic DNA was spiked with 1 ng of unmethylated lambda DNA (Promega), and the mixture was bisulfite converted. Then, the bisulfite-treated DNA was split into two portions and individually served for library preparations in forward and reverse directions. The two libraries of different directions were mixed and served for sequencing. This mixed library strategy is effective for signal complementation of biased nucleotide composition of WGBS reads^72^. Sequencing was performed with HiSeq X ten at Macrogen Japan (Tokyo, Japan), assigning a lane per sample.

We used transcriptome of WT mice under the same condition as this study obtained in our previous studies^22,23^, but instead of FPKM (fragments per kilobase of exon per million reads mapped), TPM (transcript per million) was used as the gene expression level.

We used proteome of WT mice under the same condition as this study measured with iBAQ-MS obtained in our previous study^23^.

### DMG identification

The sequenced reads were mapped on the mouse reference genome mm10 combined with the genome sequence of Escherichia phage Lambda using BMap^73^. The aligned reads were summarized with a series of in-house programs (https://github.com/FumihitoMiura/Project-2). The basic statistics of the methylome data are provided in Supplementary Data 6.

The methylation ratio of each CpG was compared between the liver and skeletal muscle by the software RADMeth^74^. CpGs satisfying q < 0.01 were defined as differentially methylated CpGs (DMCpG). Genes with at least one DMCpG at 200 bp upstream ~ 400 bp downstream of the TSS were defined as Differentially Methylated Genes (DMGs). All q values here are the p values corrected for multiple testing using the Storey method^75^. Ensembl transcript IDs are used for the genes.

### DEG identification

Genes that were not expressed in either liver or skeletal muscle were excluded by edgeR^76^. Here, expressed genes are those whose reads are present in at least 6 replicates out of 11 in either liver or skeletal muscle. The expression levels between the liver and skeletal muscle were tested against this gene set by edgeR^76^, and genes satisfying q < 0.01 were defined as differentially expressed genes (DEGs). The q values here are all p-values corrected for multiple testing using the Benjamini–Hochberg method. Ensembl transcript IDs are used for genes.

### DEP and DRP identification

Each protein was considered expressed if an iBAQ value for its expression level was obtained in at least 3 out of 5 replicates. For the 2741 proteins expressed in at least one of the organs, DEPs were identified by the following method. For proteins expressed in both tissues, iBAQ values were normalized by variance stabilized normalization^77^. Briefly, the expression amount $x$ was converted with $h(x)=\gamma \mathrm{arcsinh}(a+bx)$, where $a, b, \gamma$ were estimated from data using a robust variant of maximum likelihood estimation. Normalized expression levels were compared by limma^78^, and proteins with q < 0.01 were defined as DEP. Proteins expressed only in one tissue were also included in the DEPs.

The protein/mRNA ratio in skeletal muscle was plotted against the protein/mRNA ratio of each protein in the liver (log ratio of gene expression (TPM) to protein abundance (iBAQ value)). Linear regression was used to regress the log ratio in the liver against the log ratio in the skeletal muscle. In this regression, a protein that deviates significantly from the regression line (such that the mean squared error (MSE) decreases even slightly when the protein is excluded) is considered to have a higher protein/mRNA ratio in one tissue than in the other (Fig. 3g).

Ensembl protein IDs are used for proteins. For overlap between DEP and DMG, DEG, and DTG, Ensembl protein IDs and Ensembl transcript IDs are converted to Ensembl genes IDs. If two genes or proteins have the same Ensembl gene ID, they are overlapped.

### DTG identification

From the ChIP-atlas Peak Browser^30^, the position of the ChIP-seq peak (Threshold for significance > 500) for each TF (liver: 90 TF, skeletal muscle: 68) in liver and skeletal muscle was retrieved. When the peak of ChIP-seq of TF was located at 1000 bp upstream ~ 1000 bp downstream, TF was considered to be bound to the gene.

In the following, we used the binding status of the following TFs: TFs for which ChIP-seq data exist in both liver and skeletal muscle (Brd4, Cebpb, Ctcf, Rest, Srf, Tcf3); TFs for which ChIP-seq was performed only in the liver and not expressed in skeletal muscle (TPM = 0) (Fox1, Nr0b2, Onecut1); TFs for which ChIP-seq was performed only in skeletal muscle and not expressed in the liver (Fosl1, Myf5, Myod1, Myog, Pax3, Pax7). Each TF-binding state has a group of genes with that binding state. The distribution of the expression levels of those binding gene groups is considered to be the extent to which the TF binding state affects the expression levels. Differences in the effects of the different binding states are determined by a comparison test of the expression levels of the binding gene groups (two-tailed Welch's t-test, q < 0.01). A gene with a different TF-binding state between the liver and skeletal muscle is then defined as a Different TF-bound gene (DTG) if it is significant in this comparison test. Both q-values here are p-values corrected for multiple testing using the Storey method.

In addition to the DTGs identified above, the following binding states with different effects on expression levels were inferred from the test results and included in the DTGs. If the difference in the TF-binding status of a gene consists only of combinations of TFs that are significantly different in the comparison test, we infer that the gene is DTG.

All q values here are p values corrected for multiple testing using the Storey method. Ensembl transcript IDs are used for genes.

### Enrichment analysis of DEGs and DEPs

We identified pathways in which DM-DEGs, DT-DEGs, DM-DEPs, DT-DEPs, or DR-DEPs were enriched (Supplementary Data 5, q < 0.01 by right-tailed Fisher's exact test). Pathways were classified by KEGG pathway class. There were five pathway classes: “Cellular process”, which is related to intracellular organelles; “Environmental information processing”, which summarizes signal transduction pathways; “Genetic information processing”, which is related to DNA replication and central dogma; “Metabolism”, which includes a metabolic pathway; “Organismal systems”, which describe the functions of organs. We used all measured genes or proteins as background. The q values here are all p-values corrected for multiple testing using the Benjamini–Hochberg method.

### Supplementary Information

Supplementary Information 1.

Supplementary Information 2.

Supplementary Information 3.

Supplementary Information 4.

Supplementary Information 5.

Supplementary Information 6.

Supplementary Information 7.

Supplementary Information 8.

## Data Availability

The omic datasets generated in this study are uploaded into Supplemental Data 1–3. WGBS reads measured in this study has been deposited in the DNA DataBank of Japan Sequence Read Archive (JSRA) (www.ddbj.nig.ac.jp/) with project Accession Number DRA016209. The aligned reads were summarized with a series of in-house programs (https://github.com/FumihitoMiura/Project-2).

## Code availability

The code used for the analysis in this paper is available from corresponding author upon request.
