# Supplementary material for: DNA hypomethylation characterizes genes encoding tissue-dominant functional proteins in liver and skeletal muscle
Source: Sci Rep. 2023 Nov 5;13:19118. doi: 10.1038/s41598-023-46393-5 (PMC10625943; doi:10.1038/s41598-023-46393-5)
Supplement: Supplementary file 1 — Supplementary Information 1. [file 41598_2023_46393_MOESM1_ESM.pdf]

Title

DNA hypomethylation characterizes genes encoding tissue-dominant functional proteins in liver and skeletal muscle

Authors

Hideki Maehara[1], Toshiya Kokaji[1,2], Atsushi Hatano[1,3], Yutaka Suzuki[4], Masaki Matsumoto[3], Keiichi I. Nakayama[5], Riku Egami[4], Takaho Tsuchiya[6,7], Haruka Ozaki[6,7], Keigo Morita[1], Masaki Shirai[1], Dongzi Li[1], Akira Terakawa[1], Saori Uematsu[4], Ken-ichi Hironaka[1], Satoshi Ohno[1,8,9], Hiroyuki Kubota[10], Hiromitsu Araki[11], Fumihito Miura[11], Takashi Ito[11], Shinya Kuroda[1,4,8]*

Affiliations

[1]Department of Biological Sciences, Graduate School of Science, The University of Tokyo, 7-3-1 Hongo, Bunkyo-ku, Tokyo 113-0033, Japan

[2]Data Science Center, Nara Institute of Science and Technology, 8916-5 Takayama, Ikoma, Nara, Japan

[3]Department of Omics and Systems Biology, Graduate School of Medical and Dental Sciences, Niigata University, 757 Ichibancho, Asahimachi-dori, Chuo-ku, Niigata City, Niigata 951-8510, Japan

[4]Department of Computational Biology and Medical Sciences, Graduate School of Frontier Sciences, The University of Tokyo, 5-1-5 Kashiwanoha, Kashiwa, Chiba 277-8562, Japan

[5]Department of Molecular and Cellular Biology, Medical Institute of Bioregulation, Kyushu University, 3-1-1 Maidashi, Higashi-ku, Fukuoka 812-8582, Japan.

[6]Bioinformatics Laboratory, Faculty of Medicine, University of Tsukuba, Ibaraki 305-8575, Japan

[7]Center for Artificial Intelligence Research, University of Tsukuba, Ibaraki 305-8577, Japan

[8]Molecular Genetics Research Laboratory, Graduate School of Science, University of Tokyo, 7-3-1 Hongo, Bunkyo-ku, Tokyo 113-0033, Japan

[9]Department of AI Systems Medicine, M&D Data Science Center, Tokyo Medical and Dental University, Tokyo 113-8510, Japan

[10]Division of Integrated Omics, Medical Research Center for High Depth Omics, Medical Institute of Bioregulation, Kyushu University, 3-1-1 Maidashi, Higashi-ku, Fukuoka, Fukuoka 812-8582, Japan
[11]Department of Biochemistry, Kyushu University Graduate School of Medical Sciences, Fukuoka 812-8582, Japan.
*Correspondence to: skuroda@bs.s.u-tokyo.ac.jp (S.K.)

Supplementary Text

Identification of Different TF-bound Genes (DTGs) and the association between DMGs, DTGs, DEGs, DRPs, and DEPs (Supplementary Fig. 2, Supplementary Fig. 3)

Gene expression is also regulated by transcription factors (TFs). TF-binding status and DNA hypomethylation have also been shown to be interrelated[1]. We retrieved peaks for each TF (liver: 90 TFs, skeletal muscle: 68 TFs) in the liver and skeletal muscle from ChIP-atlas Peak Browser[2]. Using this TF-binding information, we examined the relationship of gene expression between DNA methylation and TF-binding status.

We examined the overlap between the TF-binding region and hypo- and hypermethylated CpGs (Supplementary Table 1). We identified TFs whose binding regions were significantly overlapped with the hypomethylated CpGs or the hypermethylated CpGs (right-tailed Fisher's exact test $q < 0.01$). The binding regions of all TFs except Cbx5 and Crtc in the liver significantly overlapped with hypomethylated CpGs (Supplementary Table 1), and no TFs overlapped with hypermethylated CpGs. All skeletal muscle TFs were significantly overlapped with hypomethylated CpG. This result suggests the possibility that the TFs preferentially bind to the hypomethylated regions, or that the TFs trigger DNA hypomethylation.

To compare the TFs between the liver and skeletal muscle, we used the TFs for which ChIP-seq data exist in both liver and skeletal muscle (Brd4, Cebpb, Ctcf, Rest, Srf, Tcf3), TFs for which ChIP-seq is performed only in the liver and not expressed in skeletal muscle (Fox1, Nr0b2, Onecut1), or TFs for which ChIP-seq was performed only in skeletal muscle and not expressed in the liver (Fosl1, Myf5, Myod1, Myog, Pax3, Pax7) (Supplementary Table 2). When a TF-binding peak was present at 1000 bp upstream ~ 1000 bp downstream of a TSS of a gene, the TF

was assumed to be bound to the gene. There are TFs known to trigger DNA hypomethylation or open chromatin. Among such TFs, Foxa1, Myod1, Ctcf, and Cebpb were identified in this analysis. Foxa1 is a major TF involved in hepatocyte specification[3], Myod1 1 is a major TF involved in muscle specification[4]. Ctcf and Cebpb are involved in chromatin remodeling[5,6]. The numbers DMGs bound by Ctcf, Foxa1, Cebpb, and Myod1 were 5,058 in the liver (83% of liver-dominant DMGs) and 1,920 in skeletal muscle (34% of skeletal muscle-dominant DMGs), respectively (Supplementary Fig. 2a-b). The binding of these TFs may trigger DNA hypomethylation of the DMGs. However, in some cases, methylation inhibited the binding of TFs such as Ctcf[7]. Further verification is needed to determine whether the binding of the TFs triggers DNA hypomethylation of the DMGs.

For each gene, we retrieved binding states of the TFs in the liver and skeletal muscle and calculated the scores of each TF-binding state based on the distribution of expression levels of the bound genes (see Methods for details). We defined genes in TF-binding status with different scores in the liver and skeletal muscle as Different TF-bound genes (DTGs). There were 3,645 (14%) DTGs activated in the liver, referred to as liver-dominant DTGs, and 6,936 (27%) DTGs activated in skeletal muscle, referred to as skeletal muscle-dominant DTGs (Supplementary Fig. 2c).

We also examined the overlaps between DTGs, DEGs, and DEPs. For the liver, among 1,374 proteins encoded by liver-dominant DEGs, 358 DEPs (18% of liver-dominant DEPs) were encoded by DT-DEGs, henceforth referred to as DT-DEPs (Supplementary Fig. 3c). For skeletal muscle, among 908 proteins encoded by skeletal muscle-dominant DEGs, 177 (35% of skeletal muscle-dominant DEPs) were DT^DEPs (Supplementary Fig. 3d).

We performed enrichment analyses of the DT-DEPs (Supplementary Fig. 3e-f). The DTDEPs were enriched in pathways belonging to the "Metabolism" and "Organismal Systems" such as complement and coagulation in the liver, and cardiac muscle contraction in skeletal muscle. These pathways in which DT-DEPs were enriched were similar to those in which DM-DEPs were enriched. We classified DT-DEPs according to which TFs were bound (Supplementary Fig. 3e-f). There are many pathways in which Cebpb-bound liver-dominant DT-DEPs (Supplementary Fig. 3e) and Brd4-bound skeletal muscle-dominant DT-DEPs are enriched (Supplementary Fig. 3f).

In summary, possible causes of differences in protein expression levels include differences in gene expression related to DNA hypomethylation, TF binding, translation and degradation. DEGs

represent the differences in expression level (Fig. 3d). DMGs represent the difference in methylation (Fig. 3b). DTGs represent the differences in TF-binding (Supplementary Fig. 2c). DRPs represent differences in post-transcriptional regulation (Fig. 3h). Among the liver-dominant DEPs, 17% were DM-DEPs, 18% were DT-DEPs, and 7% were DR-DEPs (Supplementary Fig. 3c). Among the skeletal muscle-dominant DEPs, 13% were DM-DEPs, 35% were DT-DEPs, and 14% were DR-DEPs (Supplementary Fig. 3d). Therefore, among DEPs in both tissues, approximately 15% were DM-DEPs, 20% were DT-DEPs, and 10% were DR-DEPs. More than half of the DEPs are not DM-DEPs, DT-DEPs, nor DR-DEPs. These DEPs may be regulated by epigenomes other than DNA methylation or TFs for which there are no data in this study or may be remotely regulated by enhancers.

Supplementary Figures and Supplementary Tables

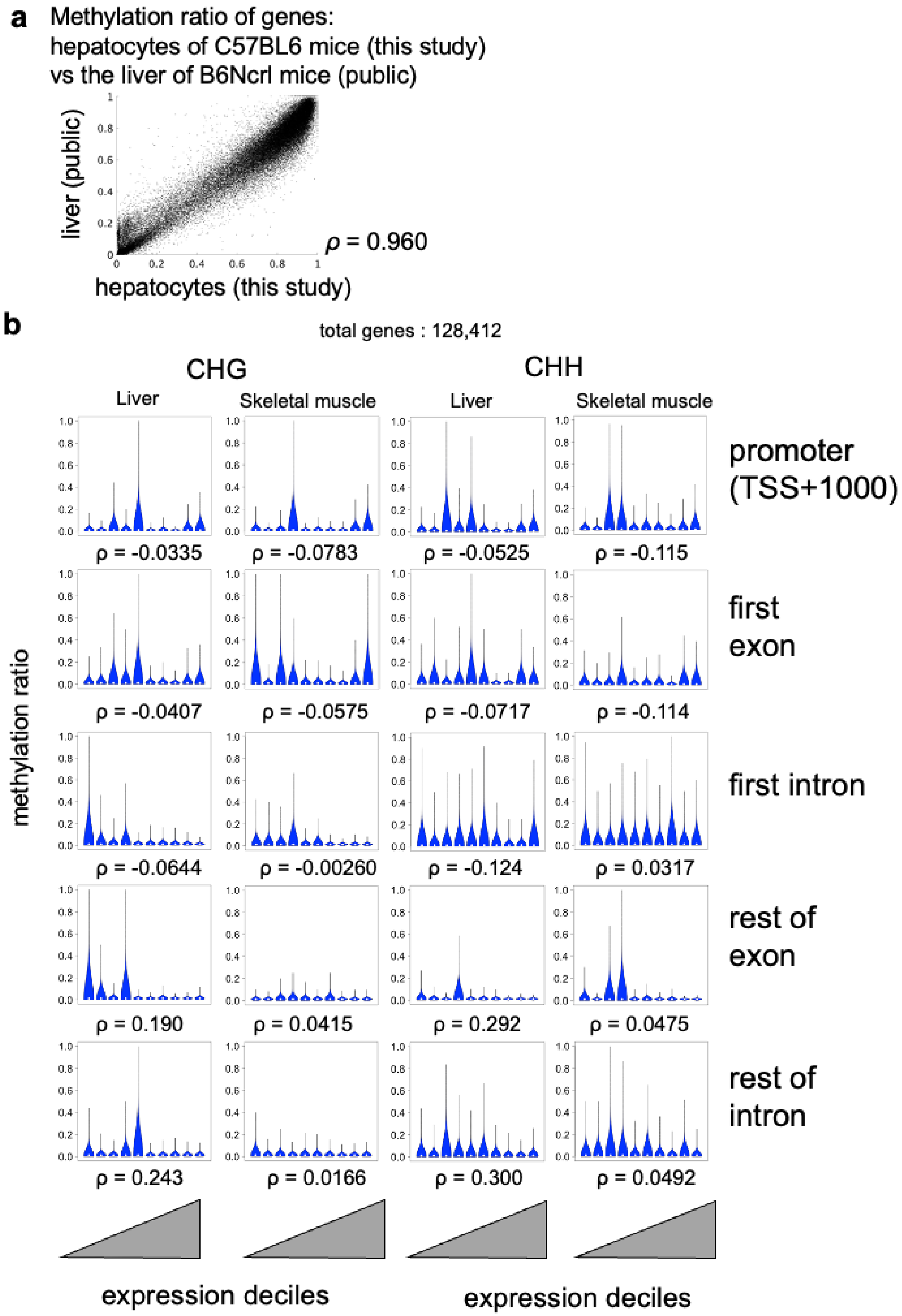
a
Methylation ratio of genes:
hepatocytes of C57BL6 mice (this study)
vs the liver of B6Ncrl mice (public)
liver (public)
hepatocytes (this study)
ρ = 0.960
b
total genes : 128,412
CHG
CHH
Liver
Skeletal muscle
Liver
Skeletal muscle
promoter (TSS+1000)
ρ = -0.0335
ρ = -0.0783
ρ = -0.0525
ρ = -0.115
first exon
ρ = -0.0407
ρ = -0.0575
ρ = -0.0717
ρ = -0.114
first intron
ρ = -0.0644
ρ = -0.00260
ρ = -0.124
ρ = 0.0317
rest of exon
ρ = 0.190
ρ = 0.0415
ρ = 0.292
ρ = 0.0475
rest of intron
ρ = 0.243
ρ = 0.0166
ρ = 0.300
ρ = 0.0492
methylation ratio
expression deciles
expression deciles

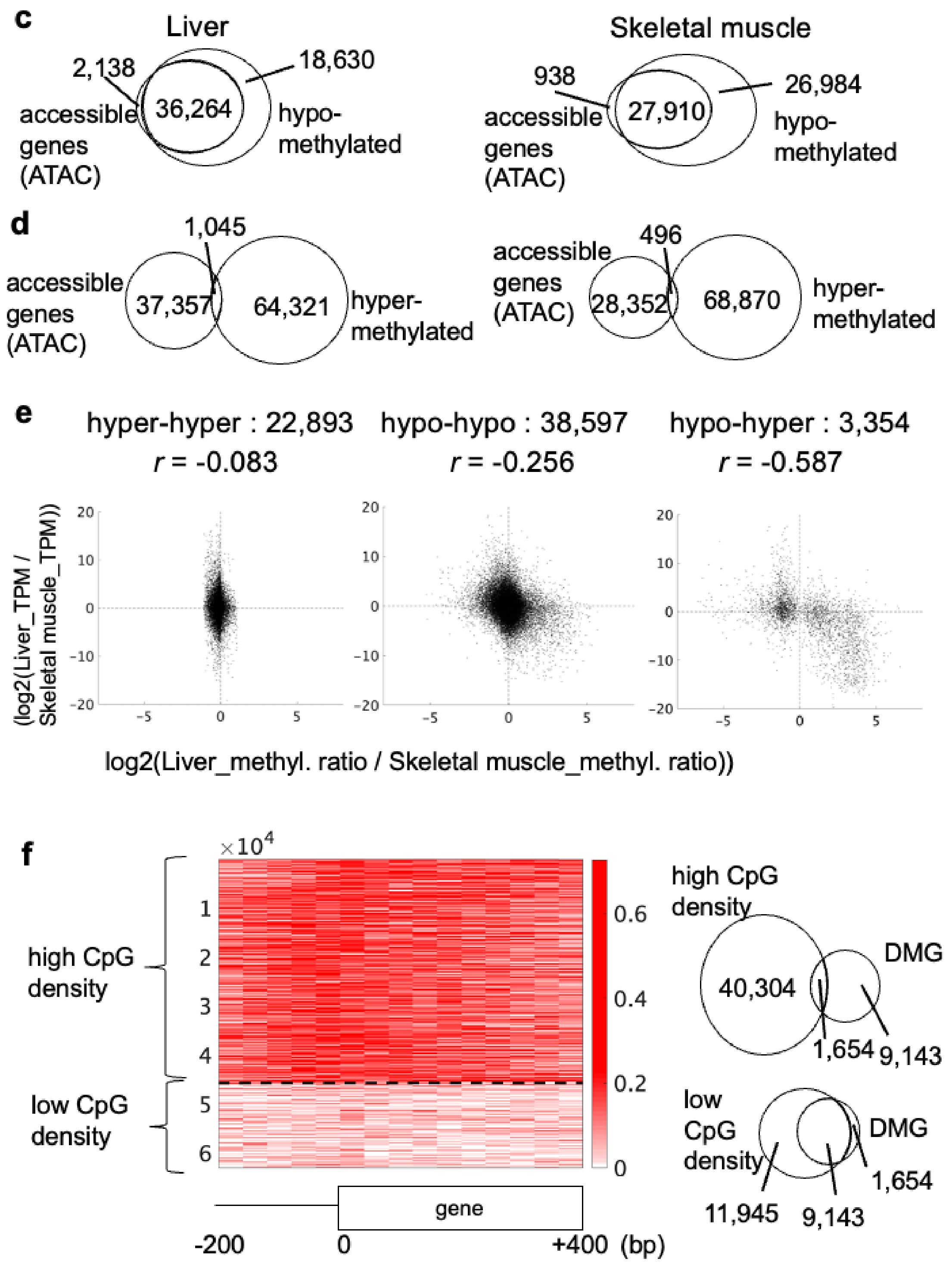
c
Liver
2,138
18,630
36,264
accessible genes (ATAC)
hypo-methylated
Skeletal muscle
938
26,984
27,910
accessible genes (ATAC)
hypo-methylated
d
1,045
accessible genes (ATAC)
37,357
64,321
hyper-methylated
496
accessible genes (ATAC)
28,352
68,870
hyper-methylated
e
hyper-hyper : 22,893
r = -0.083
hypo-hypo : 38,597
r = -0.256
hypo-hyper : 3,354
r = -0.587
(log2(Liver_TPM / Skeletal muscle_TPM))
log2(Liver_methyl. ratio / Skeletal muscle_methyl. ratio))
f
×10^4
high CpG density
low CpG density
gene
-200
0
+400 (bp)
high CpG density
40,304
DMG
1,654
9,143
low CpG density
DMG
11,945
9,143
1,654

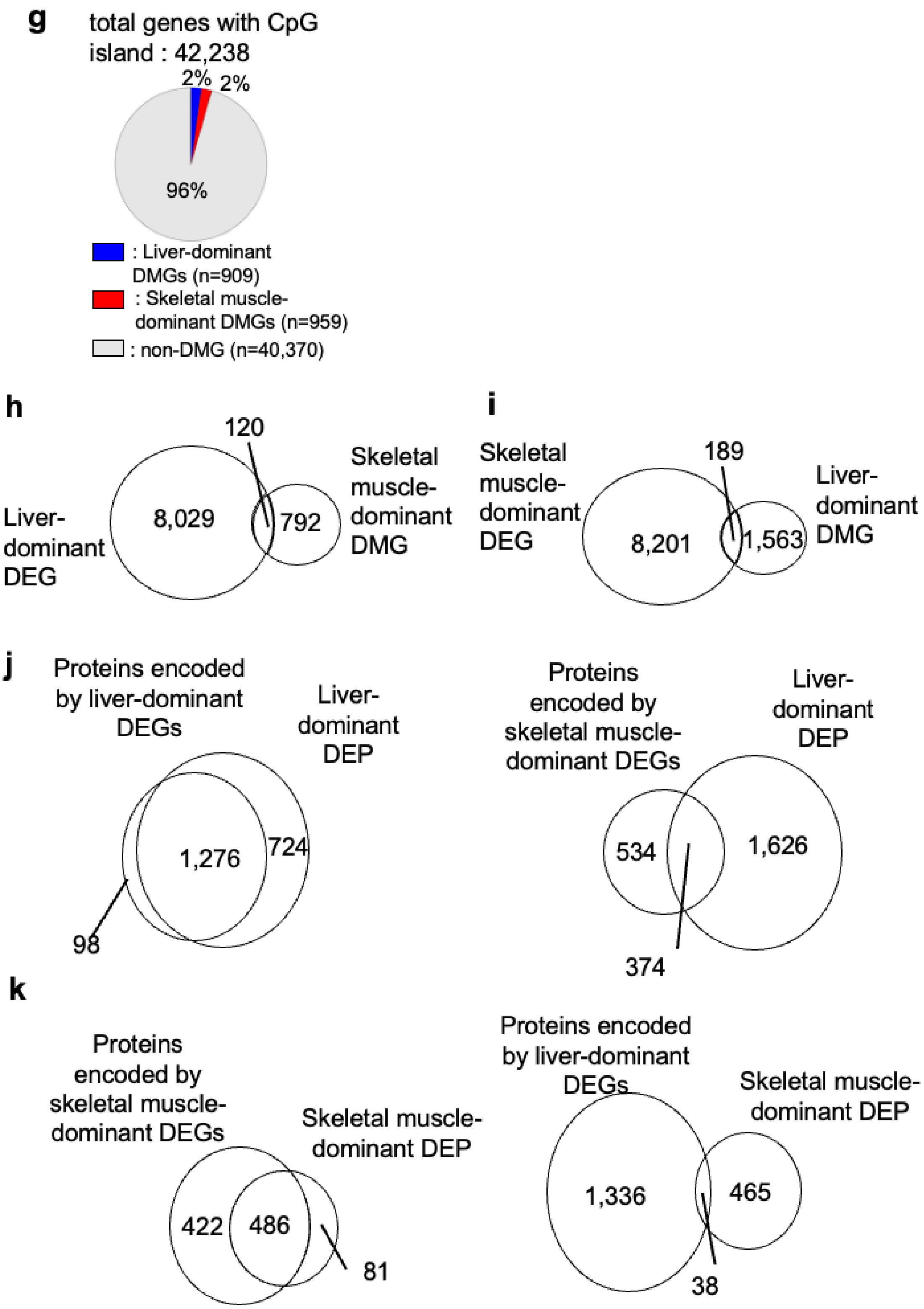
g
total genes with CpG island : 42,238
2%
2%
96%
: Liver-dominant DMGs (n=909)
: Skeletal muscle-dominant DMGs (n=959)
: non-DMG (n=40,370)
h
120
8,029
792
Liver-dominant DEG
Skeletal muscle-dominant DMG
i
189
Skeletal muscle-dominant DEG
8,201
1,563
Liver-dominant DMG
j
Proteins encoded by liver-dominant DEGs
Liver-dominant DEP
1,276
724
98
Proteins encoded by skeletal muscle-dominant DEGs
Liver-dominant DEP
534
1,626
374
k
Proteins encoded by skeletal muscle-dominant DEGs
Skeletal muscle-dominant DEP
422
486
81
Proteins encoded by liver-dominant DEGs
Skeletal muscle-dominant DEP
1,336
465
38

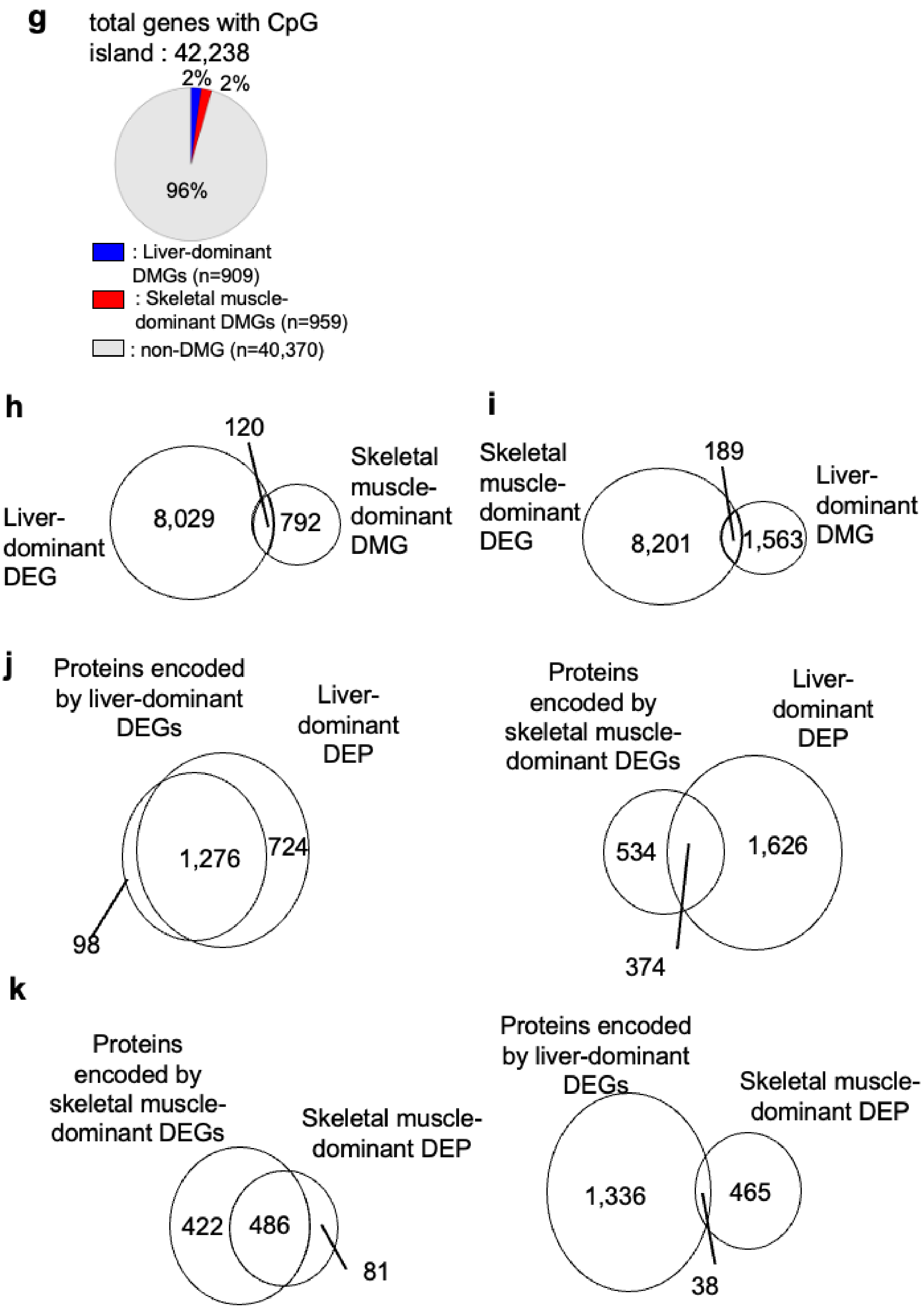
g
total genes with CpG island : 42,238
2%
2%
96%
: Liver-dominant DMGs (n=909)
: Skeletal muscle-dominant DMGs (n=959)
: non-DMG (n=40,370)
h
Liver-dominant DEG
8,029
120
792
Skeletal muscle-dominant DMG
i
Skeletal muscle-dominant DEG
8,201
189
1,563
Liver-dominant DMG
j
Proteins encoded by liver-dominant DEGs
Liver-dominant DEP
1,276
724
98
Proteins encoded by skeletal muscle-dominant DEGs
Liver-dominant DEP
534
1,626
374
k
Proteins encoded by skeletal muscle-dominant DEGs
Skeletal muscle-dominant DEP
422
486
81
Proteins encoded by liver-dominant DEGs
Skeletal muscle-dominant DEP
1,336
465
38

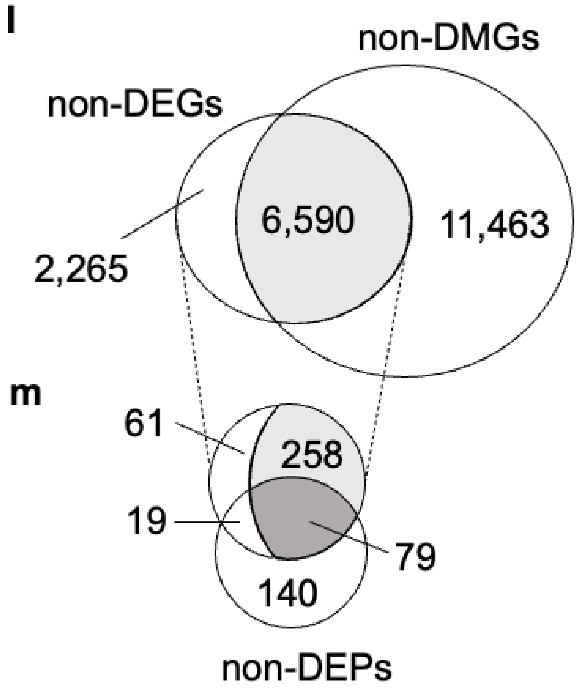

Supplementary Figure 1. Supplemental explanation of our data, DMGs, DEGs, and DEPs. (**a**) Comparison of methylation ratios (200 bp upstream ~ 400 bp downstream of TSS) of each gene between C57BL6 mouse hepatocyte in this study and B6Ncrl mouse liver in ENCODE. (**b**) Relationship between gene expression and non-CpG (CHG or CHH; H: A, C, or T) methylation across gene regions. Plots show gene expression divided into deciles from low to high and methylation ratios of non-CpG sites for each of the indicated regions of genes. Correlations between expression and methylation ratio are presented as Spearman's rank correlation coefficient ρ. (**c**) Overlap of genes in hypomethylated state and genes with ATAC-seq peaks retrieved from ChIP-atlas (open chromatin). (**d**) Overlap of genes in hypermethylated state and genes with ATAC-seq peaks (**e**) For each gene, the difference in gene expression level (log2 (TPM in liver/TPM in skeletal muscle)) was plotted against the difference in methylation ratios between liver and skeletal muscle (log2 (methylation percentage in liver/skeletal muscle)). We considered three cases: hypomethylated (0 ~ 0.4196) genes in both liver and skeletal muscle, hypomethylated genes in one tissue but hypermethylated genes in the other, and hypermethylated genes in both tissues. The number of genes in each case is shown (genes with zero expression or methylation ratio are not included because the ratio cannot be obtained). (**f**) The region near the TSS was divided into 40 bp intervals, and the density of CpG in each region was determined as (CpG number in each region / length of each region). These CpG density vectors of the genes were divided into two clusters by hierarchical clustering using Euclidean distance and Ward's method. We examined the overlap between the genes belonging to each cluster and DMGs. (**g**) DMGs among genes with CpG islands. (**h-i**) Confirm overlap for combinations of highly expressed DEGs and highly methylated DMGs. (**h**) overlap between liver-dominant DEGs and skeletal muscle-dominant DMGs. (**i**) overlap between skeletal muscle-dominant DEGs and liver-dominant DMGs. (**j-k**) Overlap for proteins encoded by DEGs and DEPs. (**j**) (left) overlap between liver-dominant DEGs and liver-dominant DEPs. (right) overlap between skeletal muscle-dominant DEGs and liver-dominant DMGs. (**k**) (left) overlap between skeletal muscle-dominant DEGs and skeletal muscle-dominant DEPs. (right) overlap between liver-dominant DEGs and skeletal muscle-dominant DEPs. (**l**) Venn diagrams show the overlap between non-DMGs and

non-DEGs. (**m**) Venn diagrams show the relationship among non-DMGs, non-DEGs, and non-DEPs.

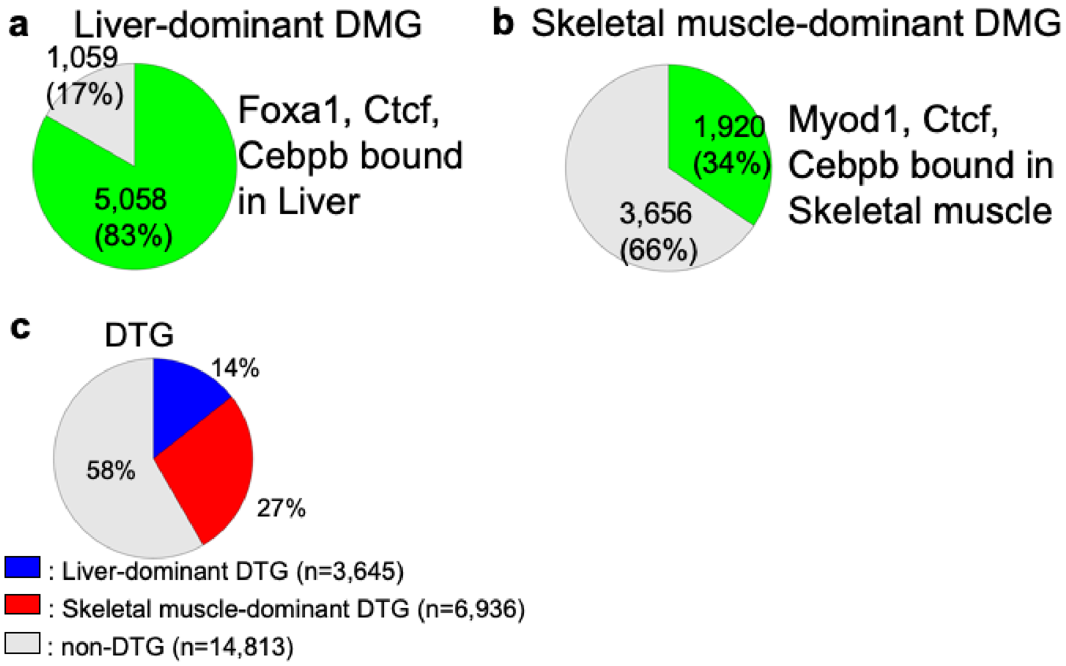

Supplementary Figure 2. Identification of DTGs and DRPs. (**a**-**b**) Proportions of genes among DMGs bound by TFs that are known to regulate DNA methylation. (**a**) liver-dominant DMG, (**b**) Skeletal muscle-dominant DMG. (**c**) Pie chart showing the proportion of differentially TF-bound genes (DTGs) in liver or skeletal muscle. The number of genes in each category is indicated.

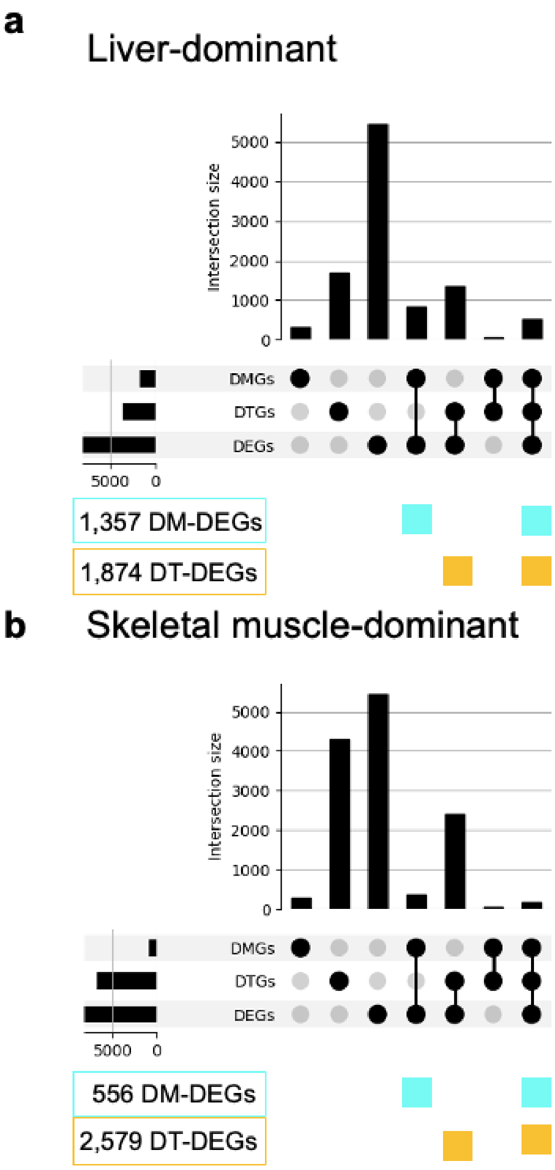
a
Liver-dominant
Intersection size
5000
4000
3000
2000
1000
0
DMGs
DTGs
DEGs
5000
0
1,357 DM-DEGs
1,874 DT-DEGs
b
Skeletal muscle-dominant
Intersection size
5000
4000
3000
2000
1000
0
DMGs
DTGs
DEGs
5000
0
556 DM-DEGs
2,579 DT-DEGs

c Liver-dominant

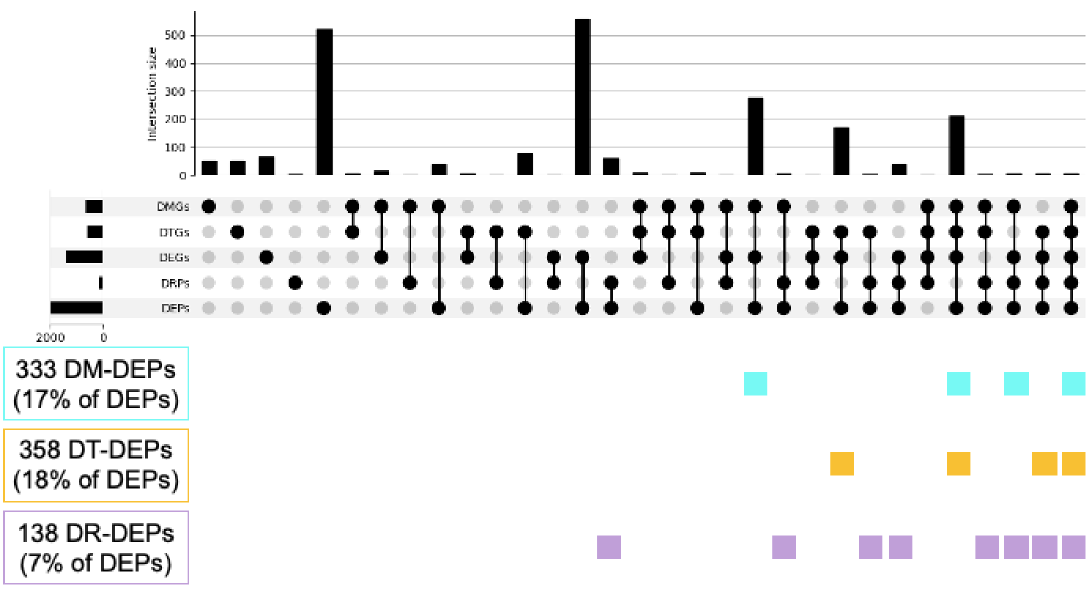

d Skeletal muscle-dominant

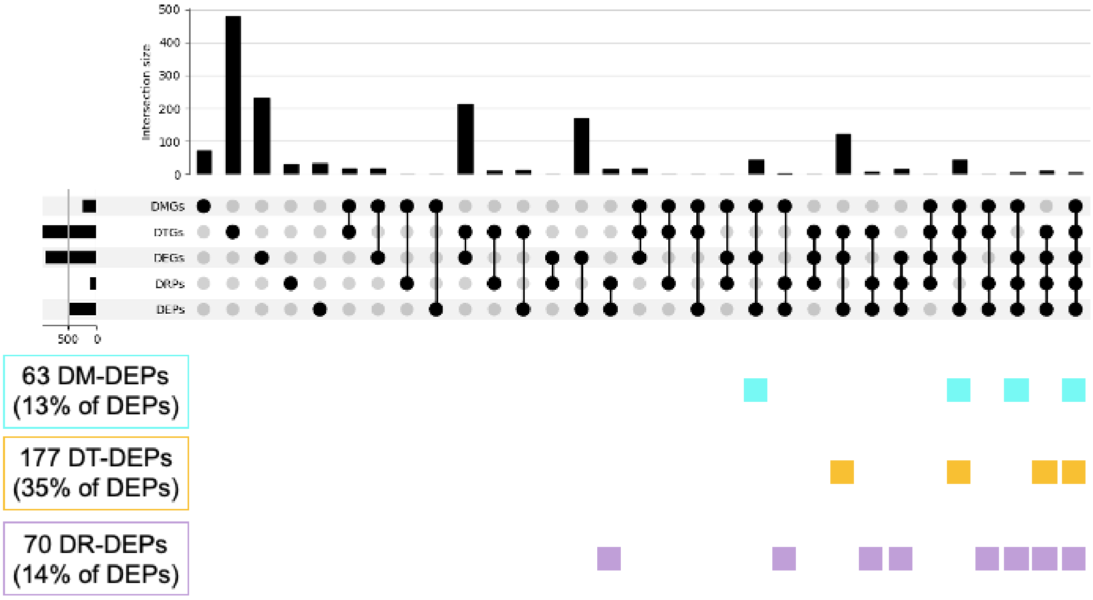

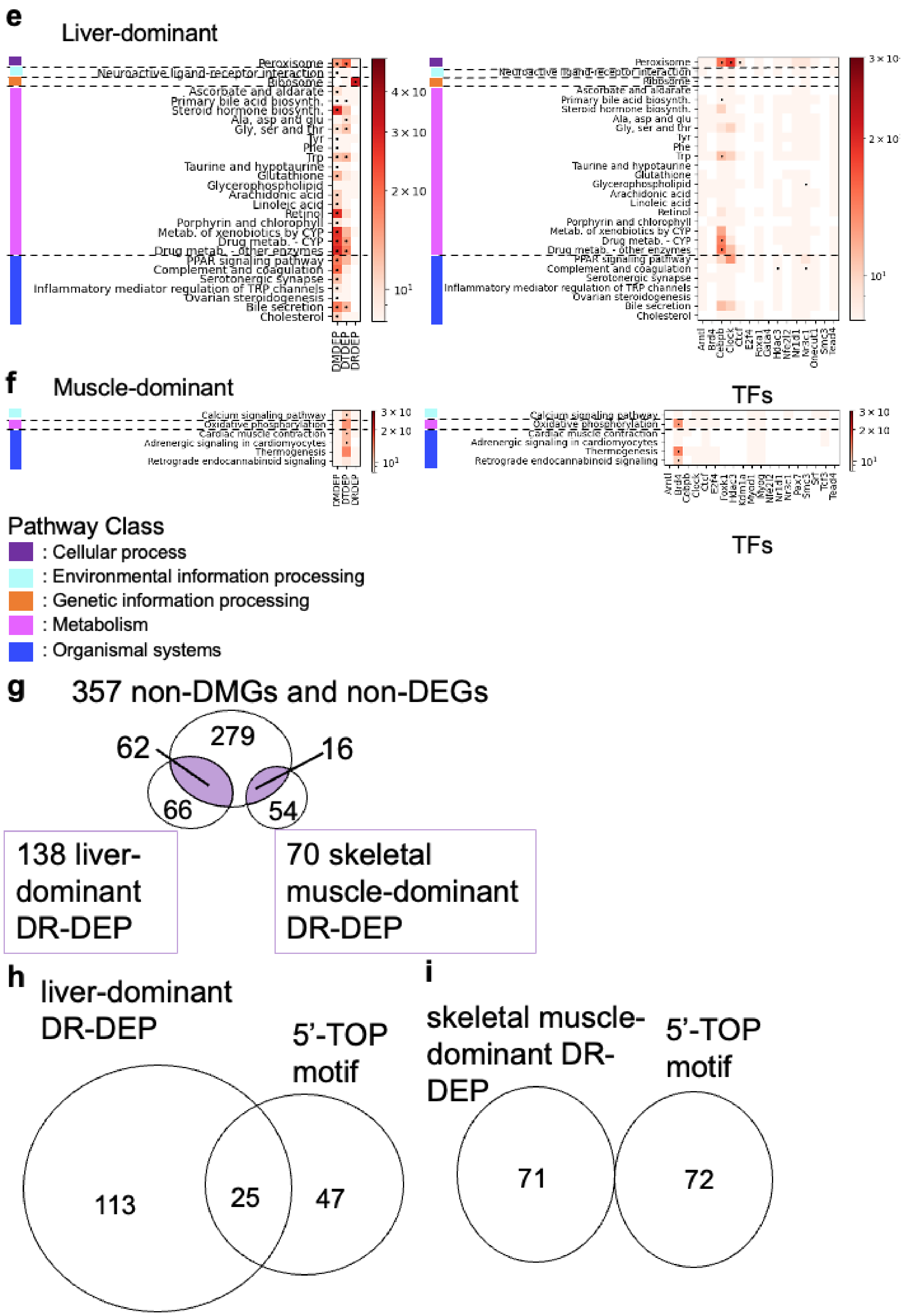
e
Liver-dominant
Peroxisome
Neuroactive ligand-receptor interaction
Ribosome
Ascorbate and aldarate
Primary bile acid biosynth.
Steroid hormone biosynth.
Ala, asp and glu
Gly, ser and thr
Tyr
Phe
Trp
Taurine and hypotaurine
Glutathione
Glycerophospholipid
Arachidonic acid
Linoleic acid
Retinol
Porphyrin and chlorophyll
Metab. of xenobiotics by CYP
Drug metab. - CYP
Drug metab. - other enzymes
PPAR signaling pathway
Complement and coagulation
Serotonergic synapse
Inflammatory mediator regulation of TRP channels
Ovarian steroidogenesis
Bile secretion
Cholesterol
DMDEP
DTDEP
DRDEP
Arntl
Brd4
Cebpb
Clock
Ctcf
E2f4
Foxa1
Gata4
Hdac3
Nfe2l2
Nr1d1
Nr3c1
Onecut1
Smc3
Tead4
TFs
f
Muscle-dominant
Calcium signaling pathway
Oxidative phosphorylation
Cardiac muscle contraction
Adrenergic signaling in cardiomyocytes
Thermogenesis
Retrograde endocannabinoid signaling
Foxk1
Hdac2
Kdm1a
Myod1
Myog
Pax7
Srf
Tcf3
TFs
Pathway Class
: Cellular process
: Environmental information processing
: Genetic information processing
: Metabolism
: Organismal systems
g
357 non-DMGs and non-DEGs
62
279
16
66
54
138 liver-dominant DR-DEP
70 skeletal muscle-dominant DR-DEP
h
liver-dominant DR-DEP
5'-TOP motif
113
25
47
i
skeletal muscle-dominant DR-DEP
5'-TOP motif
71
72

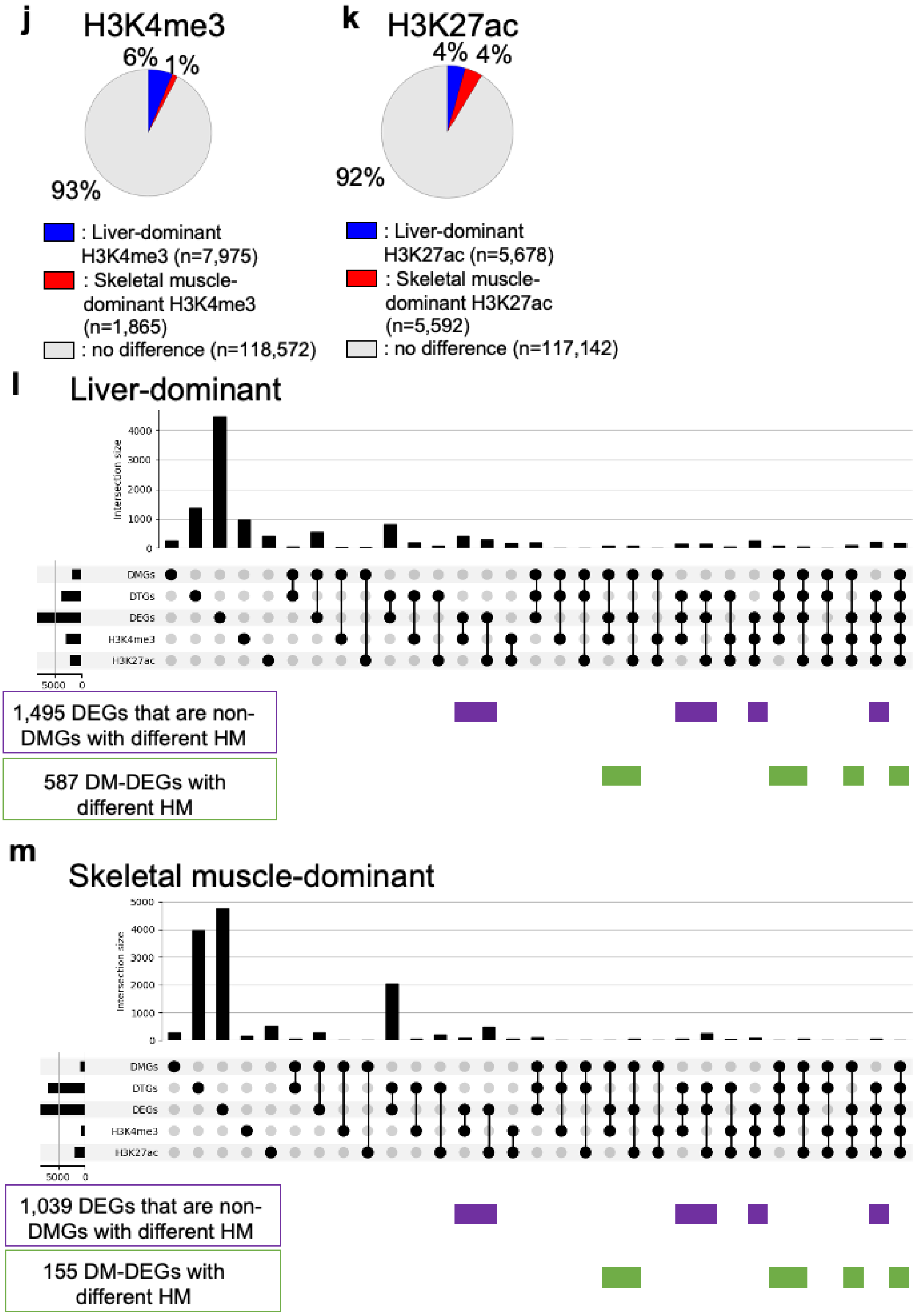
j
H3K4me3
6%
1%
93%
: Liver-dominant H3K4me3 (n=7,975)
: Skeletal muscle-dominant H3K4me3 (n=1,865)
: no difference (n=118,572)
k
H3K27ac
4%
4%
92%
: Liver-dominant H3K27ac (n=5,678)
: Skeletal muscle-dominant H3K27ac (n=5,592)
: no difference (n=117,142)
l
Liver-dominant
Intersection size
DMGs
DTGs
DEGs
H3K4me3
H3K27ac
1,495 DEGs that are non-DMGs with different HM
587 DM-DEGs with different HM
m
Skeletal muscle-dominant
Intersection size
DMGs
DTGs
DEGs
H3K4me3
H3K27ac
1,039 DEGs that are non-DMGs with different HM
155 DM-DEGs with different HM

Supplementary Figure 3. Overlaps of DMGs, DTGs, DEGs, DRPs, and DEPs. (**a-b**) The UpSet plot displays the relationships between three gene sets: DMGs, DTGs, and DEGs. The vertical axis shows the sets, and the horizontal axis shows the number of genes in each intersection. The squares under the UpSet plot columns indicate that the intersection corresponds to DM-DEGs or DT-DEGs (cyan: DM-DEGs, orange: DT-DEGs). (**a**) Liver-dominant, (**b**) Skeletal muscle-dominant, (**c-d**) The UpSet plot displays the relationships between five protein sets: proteins encoded by DMGs, proteins encoded by DTGs, proteins encoded by DEGs, DRPs, and DEPs. The vertical axis shows the sets, and the horizontal axis shows the number of proteins in each intersection. The squares under the upset plot columns indicate that the intersection corresponds to DM-DEPs, DT-DEPs, or DR-DEPs (cyan: DM-DEPs, orange: DT-DEPs, purple: DR-DEPs). (**c**) Liver-dominant, (**d**) Skeletal muscle-dominant. (**e-f**) The numbers of DM-DEPs, DT-DEPs, and DR-DEPs are shown in the heatmap (left). The number of DT-DEPs classified by the TFs is also shown (right); pathways in which DM-DEPs/DT-DEPs/DR-DEPs are enriched ($q < 0.01$ in the right-tailed Fisher's exact test) are marked with *. (**e**) Liver-dominant, (**f**) Skeletal muscle-dominant. (**g**) Overlaps between DR-DEPs and genes which were non-DMGs and non-DEGs.(metab.: metabolism, degrdn.: degradation, ER: endoplasmic reticulum, CYP: cytochrome P450, conv.: conversion, synth.: synthesis, metab. and cascades at the end of pathway names are omitted). (**h-i**) Overlaps between DR-DEPs and proteins with 5'-TOP motifs. (**h**) Liver-dominant, (**i**) Skeletal muscle-dominant. (**j-k**) Pie chart showing the proportion of differential histone modification states in liver or skeletal muscle. (**j**) H3K4me3, (**k**) H3K27ac, (**l-m**) The UpSet plot displays the relationships between five gene sets: DMGs, DTGs, DEGs, genes with different H3K4me3 states, and genes with different H3K27ac states. The vertical axis shows the sets, and the horizontal axis shows the number of proteins in each intersection. (**l**) Liver-dominant, (**m**) Skeletal muscle-dominant.

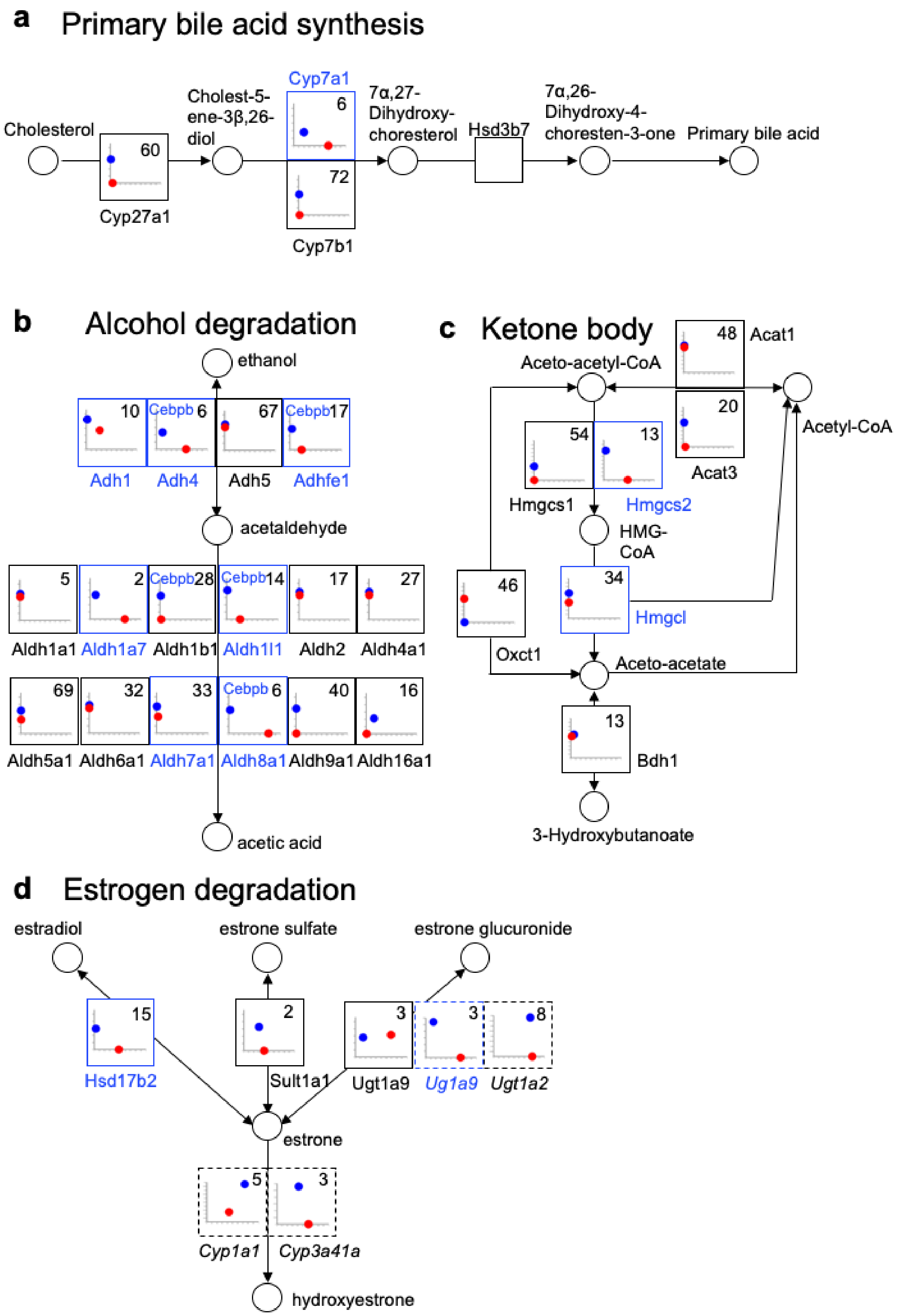
a Primary bile acid synthesis
Cholesterol
Cholest-5-ene-3β,26-diol
Cyp7a1
6
60
Cyp27a1
72
Cyp7b1
7α,27-Dihydroxy-choresterol
Hsd3b7
7α,26-Dihydroxy-4-choresten-3-one
Primary bile acid
b Alcohol degradation
ethanol
10
Adh1
Cebpb 6
Adh4
67
Adh5
Cebpb 17
Adhfe1
acetaldehyde
5
Aldh1a1
2
Aldh1a7
Cebpb 28
Aldh1b1
Cebpb 14
Aldh1l1
17
Aldh2
27
Aldh4a1
69
Aldh5a1
32
Aldh6a1
33
Aldh7a1
Cebpb 6
Aldh8a1
40
Aldh9a1
16
Aldh16a1
acetic acid
c Ketone body
Aceto-acetyl-CoA
48
Acat1
20
Acat3
Acetyl-CoA
54
Hmgcs1
13
Hmgcs2
HMG-CoA
46
Oxct1
34
Hmgcl
Aceto-acetate
13
Bdh1
3-Hydroxybutanoate
d Estrogen degradation
estradiol
estrone sulfate
estrone glucuronide
15
Hsd17b2
2
Sult1a1
3
Ugt1a9
3
Ug1a9
8
Ugt1a2
estrone
5
Cyp1a1
3
Cyp3a41a
hydroxyestrone

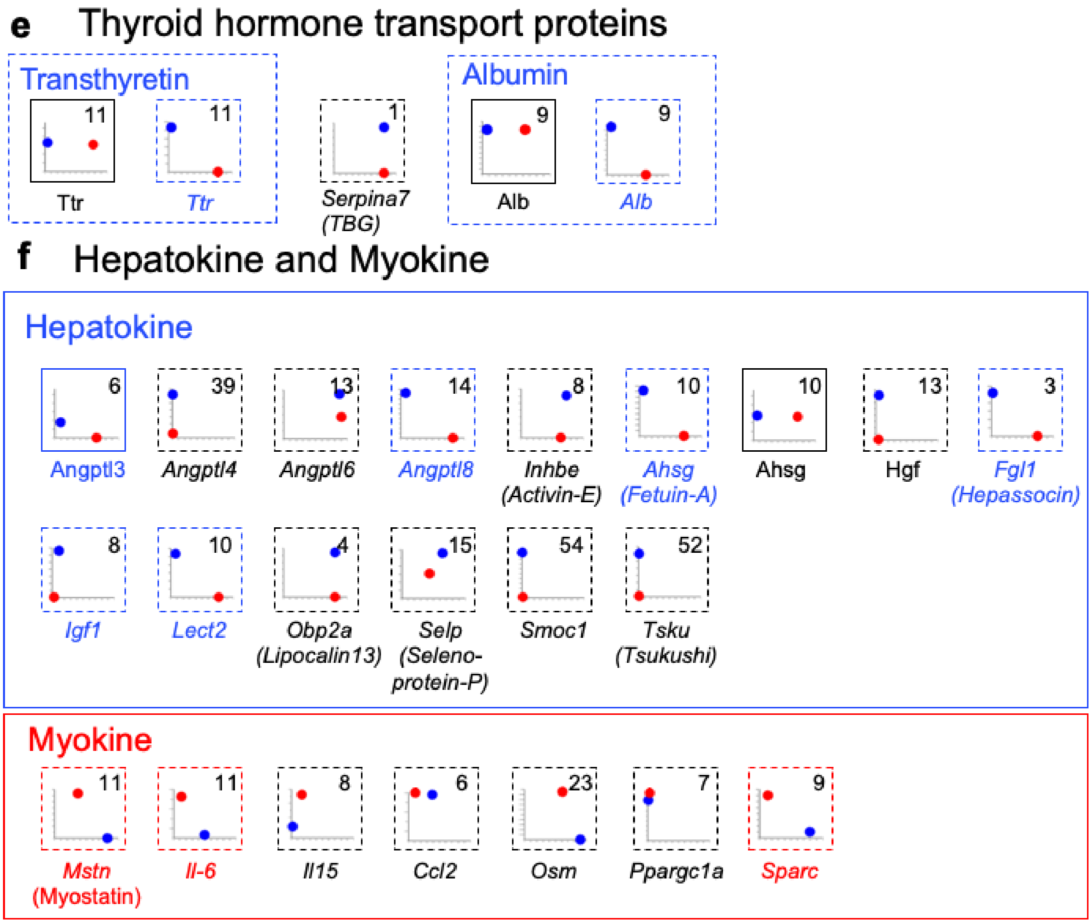

Supplementary Figure 4. Protein expression levels and methylation ratios of enzymes of pathways in the liver and muscle other than Fig. 6. Plots of expression levels versus methylation ratios for encoding genes are shown using the same rules as in Fig. 5 (blue: liver-dominant, red: skeletal muscle-dominant). (**a**) primary bile acid synthesis: a key enzyme Cyp3a1 was DMDEP. (**b**) alcohol degradation, which is a two-step reaction catalyzed by Adh and Aldh, both of which contained DMDEPs (**c**) ketone body synthesis: Hmgcs2 and Hmgcl were liver-dominant DMDEPs (**d**) estrogen metabolism: Hsd17b2 and Ugfa9 were DMDEPs (**e**) Thyroid hormone transport proteins, (**f**) Hepatokine and Myokine.

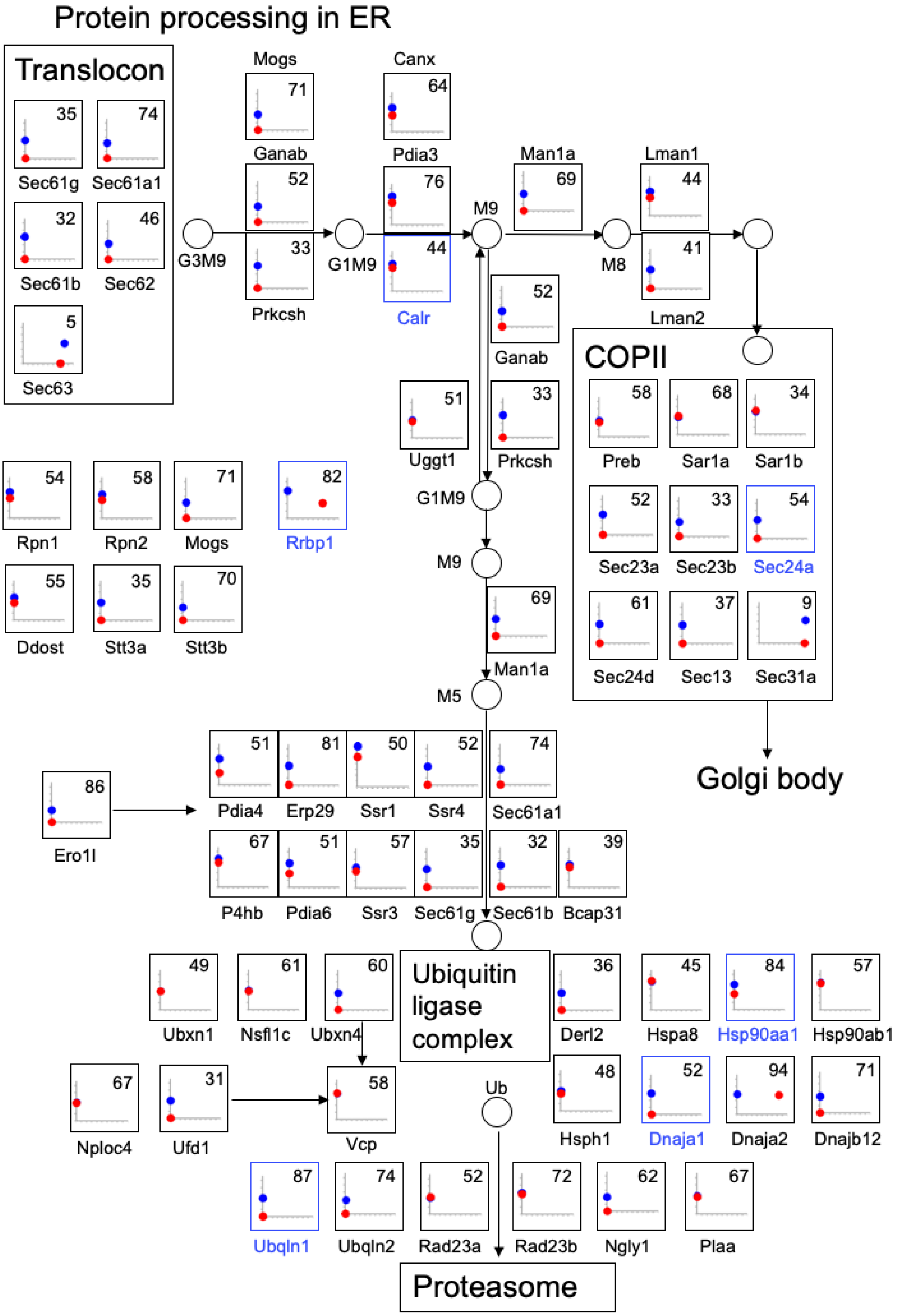

Protein processing in ER
Translocon
35
Sec61g
74
Sec61a1
32
Sec61b
46
Sec62
5
Sec63
Mogs
71
Ganab
52
33
Prkcsh
Canx
64
Pdia3
76
44
Calr
G3M9
G1M9
M9
Man1a
69
M8
Lman1
44
41
Lman2
52
Ganab
51
Uggt1
33
Prkcsh
COPII
58
Preb
68
Sar1a
34
Sar1b
52
Sec23a
33
Sec23b
54
Sec24a
61
Sec24d
37
Sec13
9
Sec31a
Golgi body
54
Rpn1
58
Rpn2
71
Mogs
82
Rrbp1
55
Ddost
35
Stt3a
70
Stt3b
G1M9
M9
69
Man1a
M5
86
Ero1l
51
Pdia4
81
Erp29
50
Ssr1
52
Ssr4
74
Sec61a1
67
P4hb
51
Pdia6
57
Ssr3
35
Sec61g
32
Sec61b
39
Bcap31
49
Ubxn1
61
Nsfl1c
60
Ubxn4
Ubiquitin ligase complex
36
Derl2
45
Hspa8
84
Hsp90aa1
57
Hsp90ab1
67
Nploc4
31
Ufd1
58
Vcp
Ub
48
Hsph1
52
Dnaja1
94
Dnaja2
71
Dnajb12
87
Ubqln1
74
Ubqln2
52
Rad23a
72
Rad23b
62
Ngly1
67
Plaa
Proteasome

Supplementary Figure 5. Protein expression levels and methylation ratios of enzymes in protein processing pathway. Plots of expression levels versus methylation ratios for encoding genes in Protein processing in ER are shown using the same rules as in Fig. 5 (blue: liver-dominant, red: skeletal muscle-dominant).

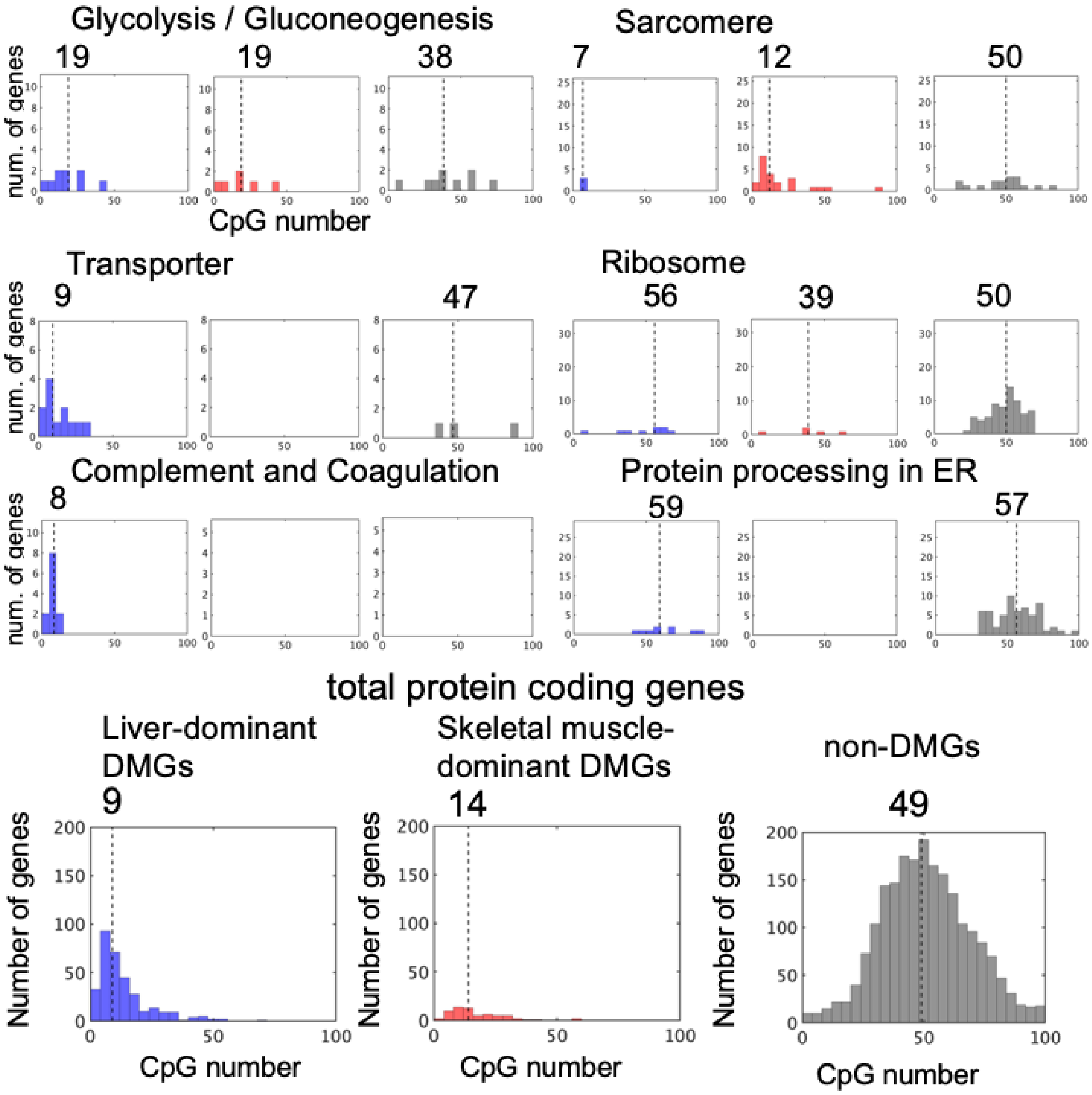

Supplementary Figure 6. Histogram of CpG numbers of DMGs, skeletal muscle-dominant DMGs, and non-DMGs in each pathway. CpG numbers of proteins encoded by liver-dominant DMGs (left column), skeletal muscle-dominant DMGs (middle column), and non-DMGs (right column) belonging to glycolysis/gluconeogenesis, transporter, complement/coagulation, sarcomere, ribosome, protein processing, and all protein-coding genes. Histograms showing the number of genes per CpG number, Bin size = 5.

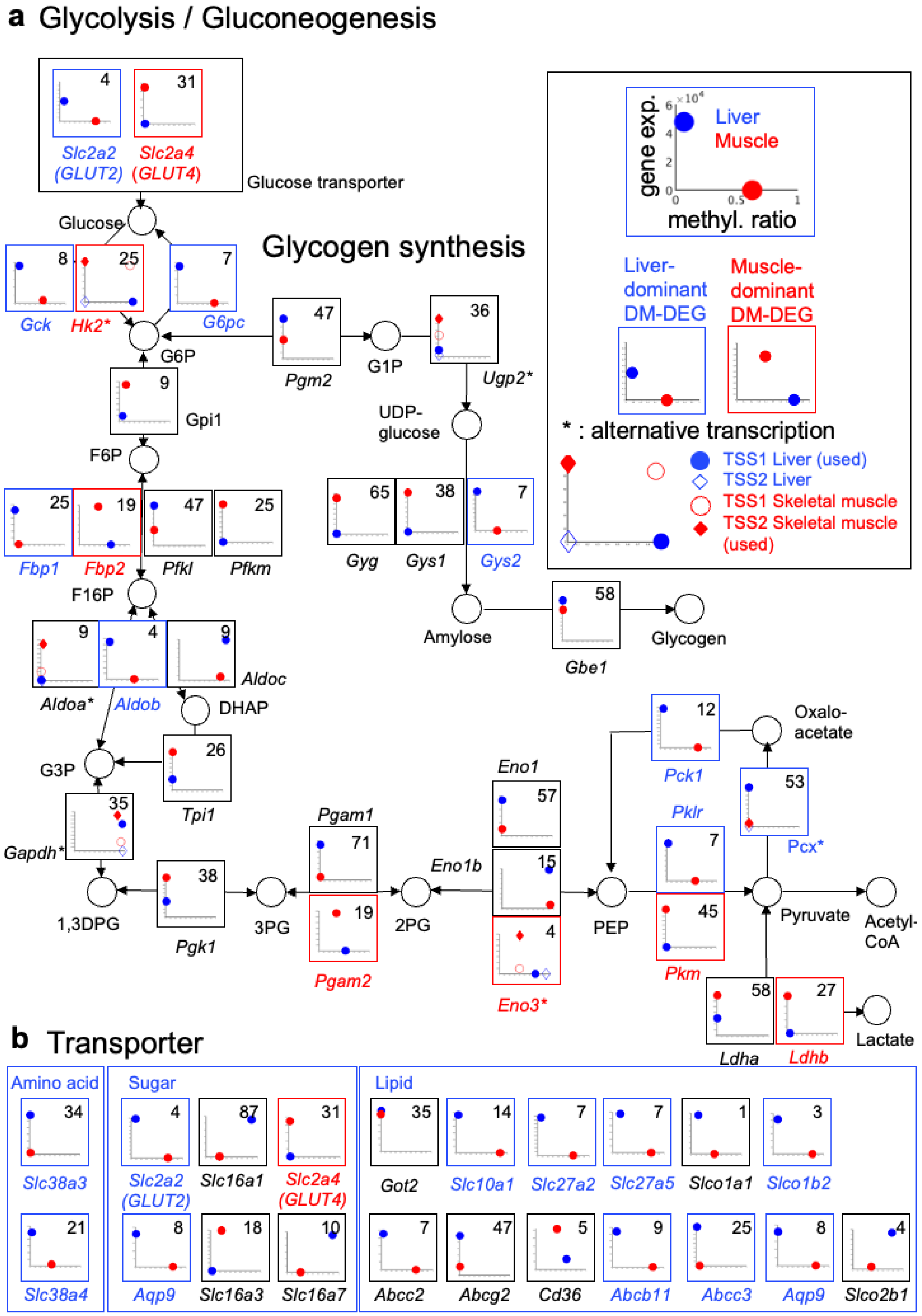
a Glycolysis / Gluconeogenesis
Glucose transporter
Slc2a2 (GLUT2)
Slc2a4 (GLUT4)
Glucose
Gck
Hk2*
G6pc
G6P
Gpi1
F6P
Fbp1
Fbp2
Pfkl
Pfkm
F16P
Aldoa*
Aldob
Aldoc
DHAP
G3P
Tpi1
Gapdh*
1,3DPG
Pgk1
3PG
Pgam1
Pgam2
2PG
Eno1
Eno1b
Eno3*
PEP
Pklr
Pkm
Pck1
Oxalo-acetate
Pcx*
Pyruvate
Acetyl-CoA
Ldha
Ldhb
Lactate
Glycogen synthesis
Pgm2
G1P
Ugp2*
UDP-glucose
Gyg
Gys1
Gys2
Amylose
Gbe1
Glycogen
gene exp.
methyl. ratio
Liver
Muscle
Liver-dominant DM-DEG
Muscle-dominant DM-DEG
* : alternative transcription
TSS1 Liver (used)
TSS2 Liver
TSS1 Skeletal muscle
TSS2 Skeletal muscle (used)
b Transporter
Amino acid
Slc38a3
Slc38a4
Sugar
Slc2a2 (GLUT2)
Slc16a1
Slc2a4 (GLUT4)
Aqp9
Slc16a3
Slc16a7
Lipid
Got2
Slc10a1
Slc27a2
Slc27a5
Slco1a1
Slco1b2
Abcc2
Abcg2
Cd36
Abcb11
Abcc3
Aqp9
Slco2b1

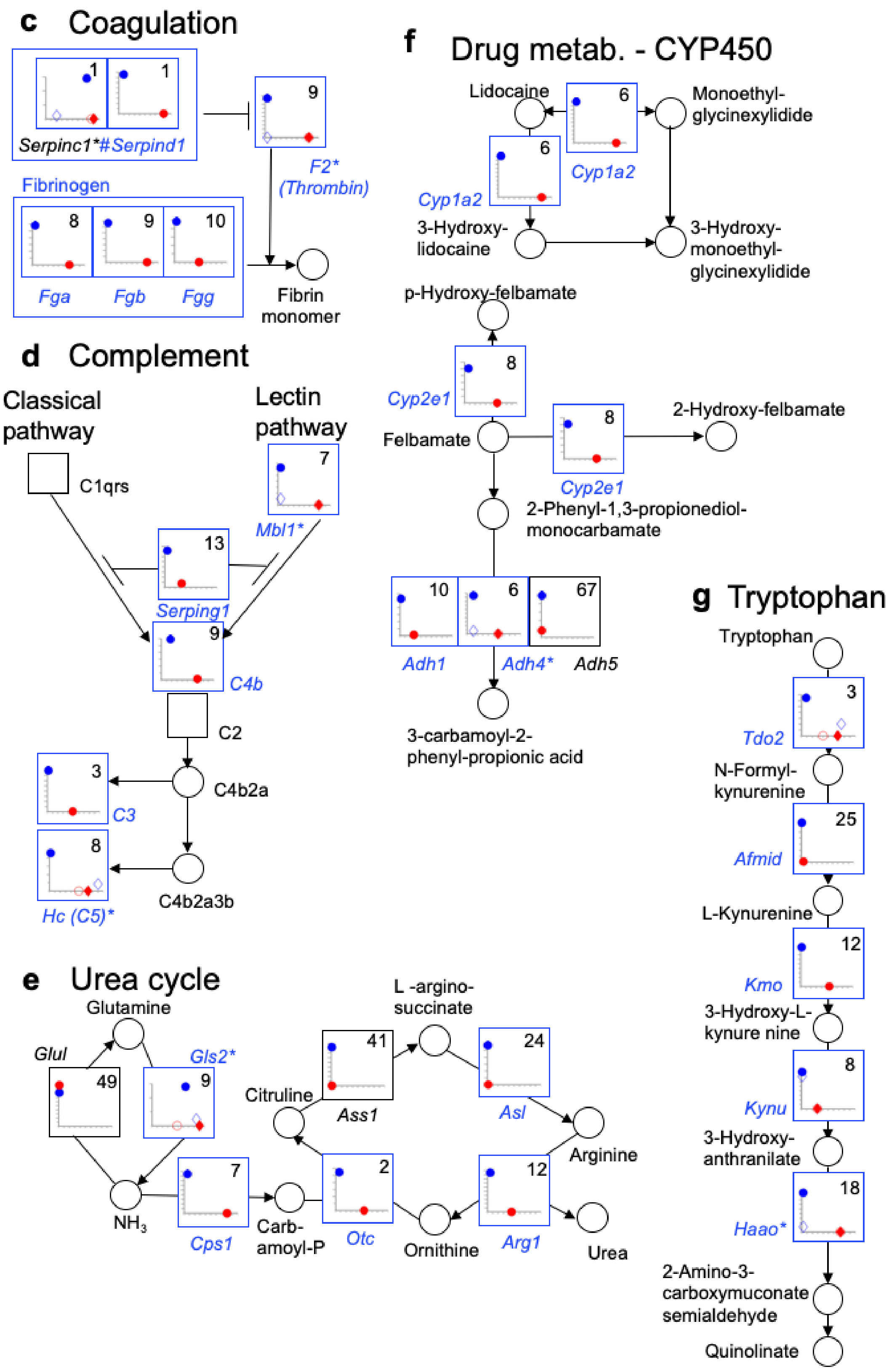
c Coagulation
Serpinc1*#Serpind1
F2* (Thrombin)
Fibrinogen
Fga Fgb Fgg
Fibrin monomer
d Complement
Classical pathway
Lectin pathway
C1qrs
Mbl1*
Serping1
C4b
C2
C4b2a
C3
Hc (C5)*
C4b2a3b
e Urea cycle
Glutamine
Glul
Gls2*
L -argino-succinate
Citrulline
Ass1
Asl
Arginine
NH3
Cps1
Carb-amoyl-P
Otc
Ornithine
Arg1
Urea
f Drug metab. - CYP450
Lidocaine
Cyp1a2
Monoethyl-glycinexylidide
3-Hydroxy-lidocaine
3-Hydroxy-monoethyl-glycinexylidide
p-Hydroxy-felbamate
Cyp2e1
Felbamate
2-Hydroxy-felbamate
2-Phenyl-1,3-propionediol-monocarbamate
Adh1 Adh4* Adh5
3-carbamoyl-2-phenyl-propionic acid
g Tryptophan
Tryptophan
Tdo2
N-Formyl-kynurenine
Afmid
L-Kynurenine
Kmo
3-Hydroxy-L-kynure nine
Kynu
3-Hydroxy-anthranilate
Haao*
2-Amino-3-carboxymuconate semialdehyde
Quinolinate

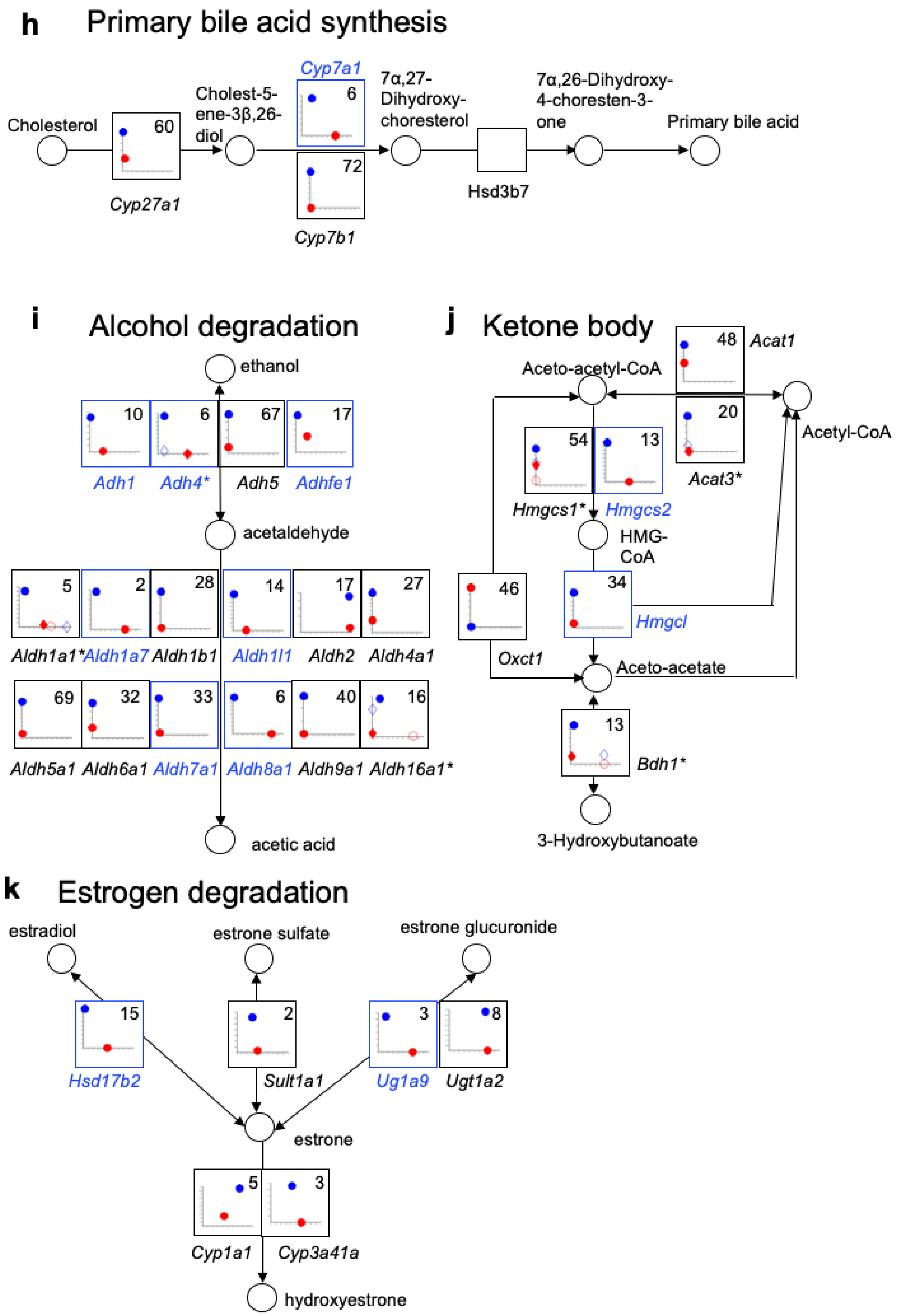
h Primary bile acid synthesis
Cholesterol
Cholest-5-ene-3β,26-diol
7α,27-Dihydroxy-choresterol
7α,26-Dihydroxy-4-choresten-3-one
Primary bile acid
Cyp27a1
Cyp7a1
Cyp7b1
Hsd3b7
60
6
72
i Alcohol degradation
ethanol
acetaldehyde
acetic acid
Adh1
Adh4*
Adh5
Adhfe1
10
6
67
17
Aldh1a1*
Aldh1a7
Aldh1b1
Aldh1l1
Aldh2
Aldh4a1
5
2
28
14
17
27
Aldh5a1
Aldh6a1
Aldh7a1
Aldh8a1
Aldh9a1
Aldh16a1*
69
32
33
6
40
16
j Ketone body
Aceto-acetyl-CoA
Acetyl-CoA
HMG-CoA
Aceto-acetate
3-Hydroxybutanoate
Acat1
Acat3*
Hmgcs1*
Hmgcs2
Hmgcl
Oxct1
Bdh1*
48
20
54
13
34
46
13
k Estrogen degradation
estradiol
estrone sulfate
estrone glucuronide
estrone
hydroxyestrone
Hsd17b2
Sult1a1
Ug1a9
Ugt1a2
Cyp1a1
Cyp3a41a
15
2
3
8
5
3

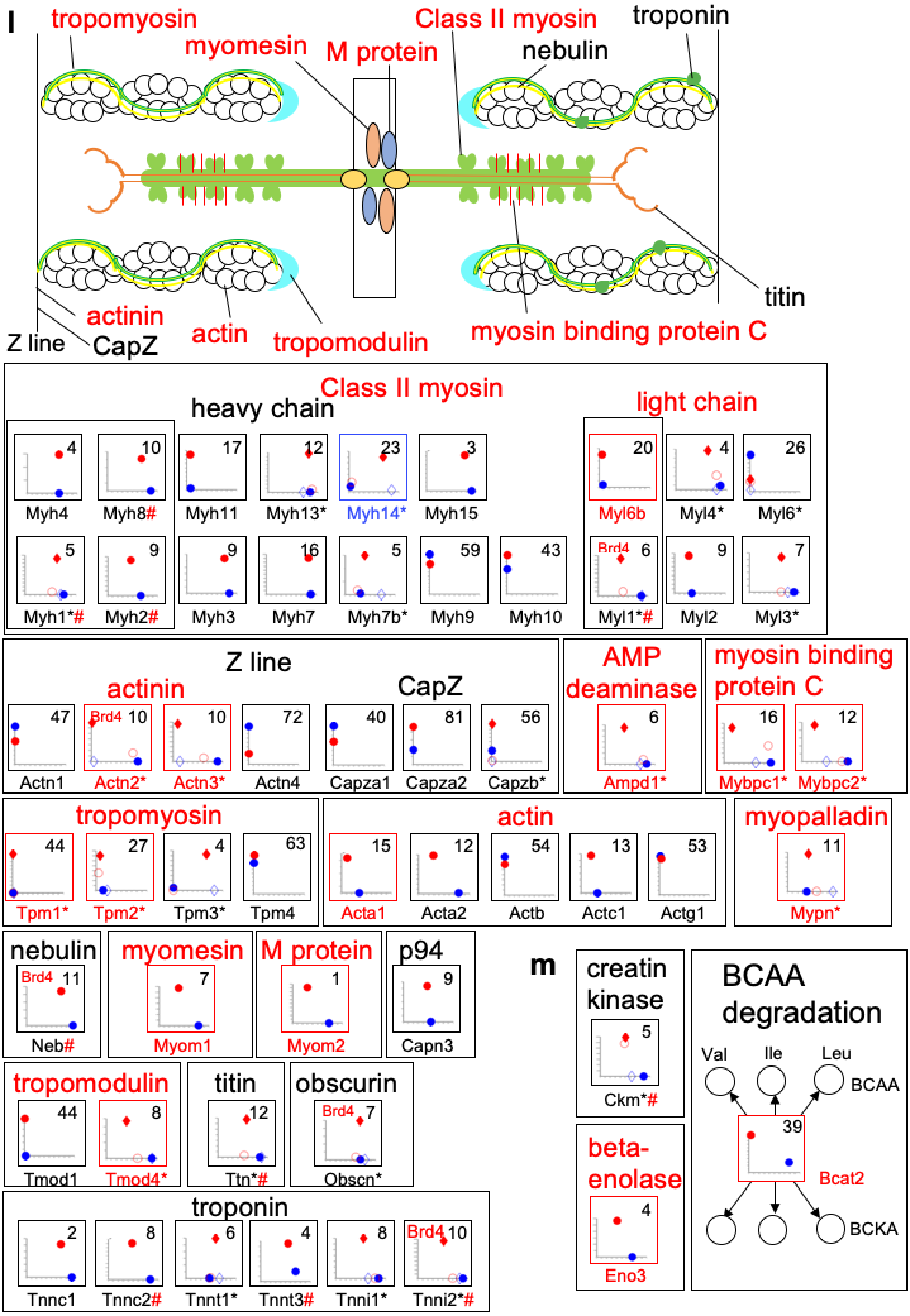
l
tropomyosin
myomesin
M protein
Class II myosin
nebulin
troponin
titin
Z line
actinin
CapZ
actin
tropomodulin
myosin binding protein C
Class II myosin
heavy chain
light chain
Myh4
Myh8#
Myh11
Myh13*
Myh14*
Myh15
Myl6b
Myl4*
Myl6*
Myh1*#
Myh2#
Myh3
Myh7
Myh7b*
Myh9
Myh10
Myl1*#
Myl2
Myl3*
Z line
actinin
CapZ
Actn1
Actn2*
Actn3*
Actn4
Capza1
Capza2
Capzb*
AMP deaminase
Ampd1*
myosin binding protein C
Mybpc1*
Mybpc2*
tropomyosin
Tpm1*
Tpm2*
Tpm3*
Tpm4
actin
Acta1
Acta2
Actb
Actc1
Actg1
myopalladin
Mypn*
nebulin
Neb#
myomesin
Myom1
M protein
Myom2
p94
Capn3
tropomodulin
Tmod1
Tmod4*
titin
Ttn*#
obscurin
Obscn*
troponin
Tnnc1
Tnnc2#
Tnnt1*
Tnnt3#
Tnni1*
Tnni2*#
m
creatin kinase
Ckm*#
beta-enolase
Eno3
BCAA degradation
Val
Ile
Leu
BCAA
Bcat2
BCKA

n Ribosome

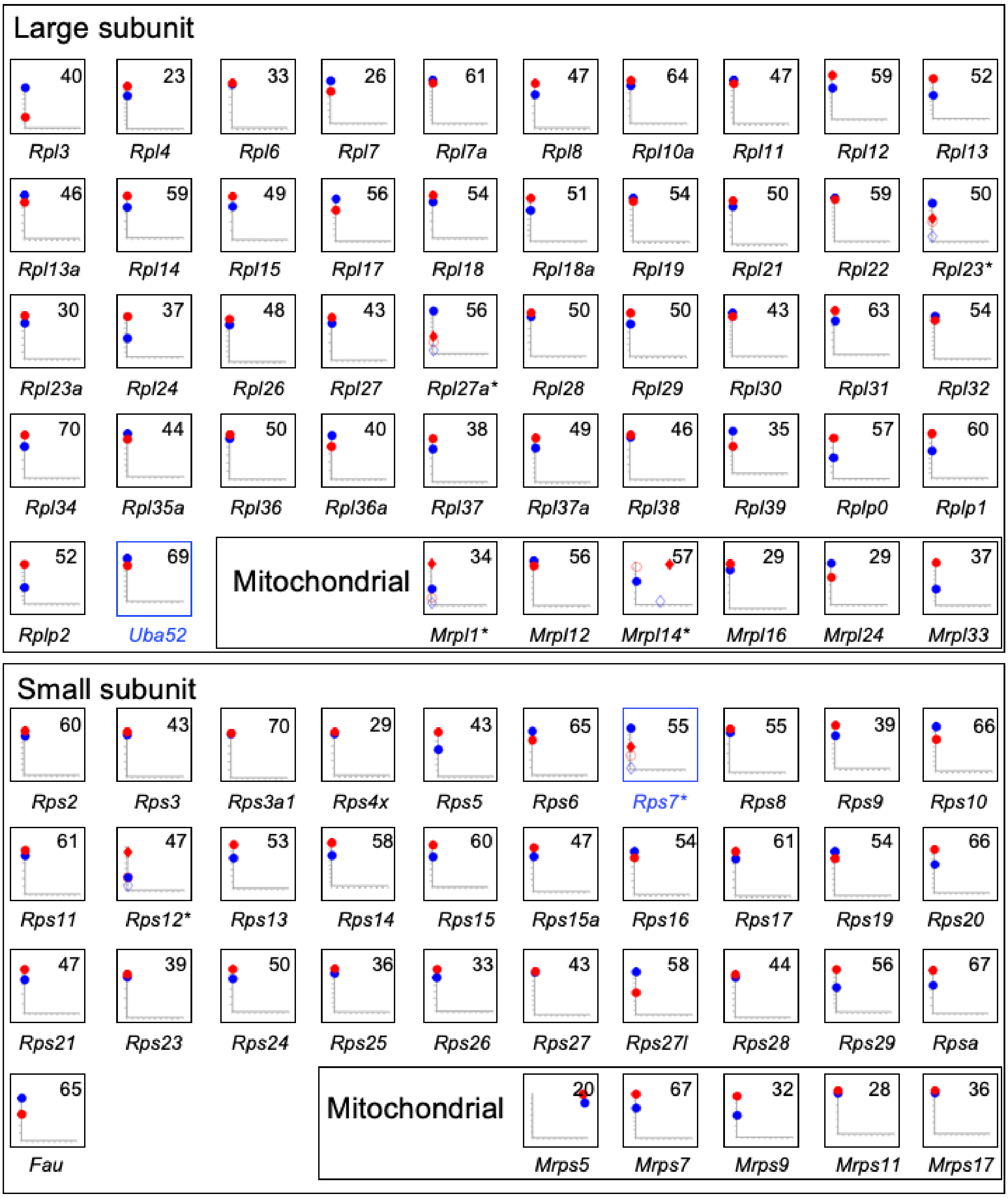
Large subunit
40 Rpl3
23 Rpl4
33 Rpl6
26 Rpl7
61 Rpl7a
47 Rpl8
64 Rpl10a
47 Rpl11
59 Rpl12
52 Rpl13
46 Rpl13a
59 Rpl14
49 Rpl15
56 Rpl17
54 Rpl18
51 Rpl18a
54 Rpl19
50 Rpl21
59 Rpl22
50 Rpl23*
30 Rpl23a
37 Rpl24
48 Rpl26
43 Rpl27
56 Rpl27a*
50 Rpl28
50 Rpl29
43 Rpl30
63 Rpl31
54 Rpl32
70 Rpl34
44 Rpl35a
50 Rpl36
40 Rpl36a
38 Rpl37
49 Rpl37a
46 Rpl38
35 Rpl39
57 Rplp0
60 Rplp1
52 Rplp2
69 Uba52
Mitochondrial
34 Mrpl1*
56 Mrpl12
57 Mrpl14*
29 Mrpl16
29 Mrpl24
37 Mrpl33
Small subunit
60 Rps2
43 Rps3
70 Rps3a1
29 Rps4x
43 Rps5
65 Rps6
55 Rps7*
55 Rps8
39 Rps9
66 Rps10
61 Rps11
47 Rps12*
53 Rps13
58 Rps14
60 Rps15
47 Rps15a
54 Rps16
61 Rps17
54 Rps19
66 Rps20
47 Rps21
39 Rps23
50 Rps24
36 Rps25
33 Rps26
43 Rps27
58 Rps27l
44 Rps28
56 Rps29
67 Rpsa
65 Fau
Mitochondrial
20 Mrps5
67 Mrps7
32 Mrps9
28 Mrps11
36 Mrps17

o Protein processing in ER

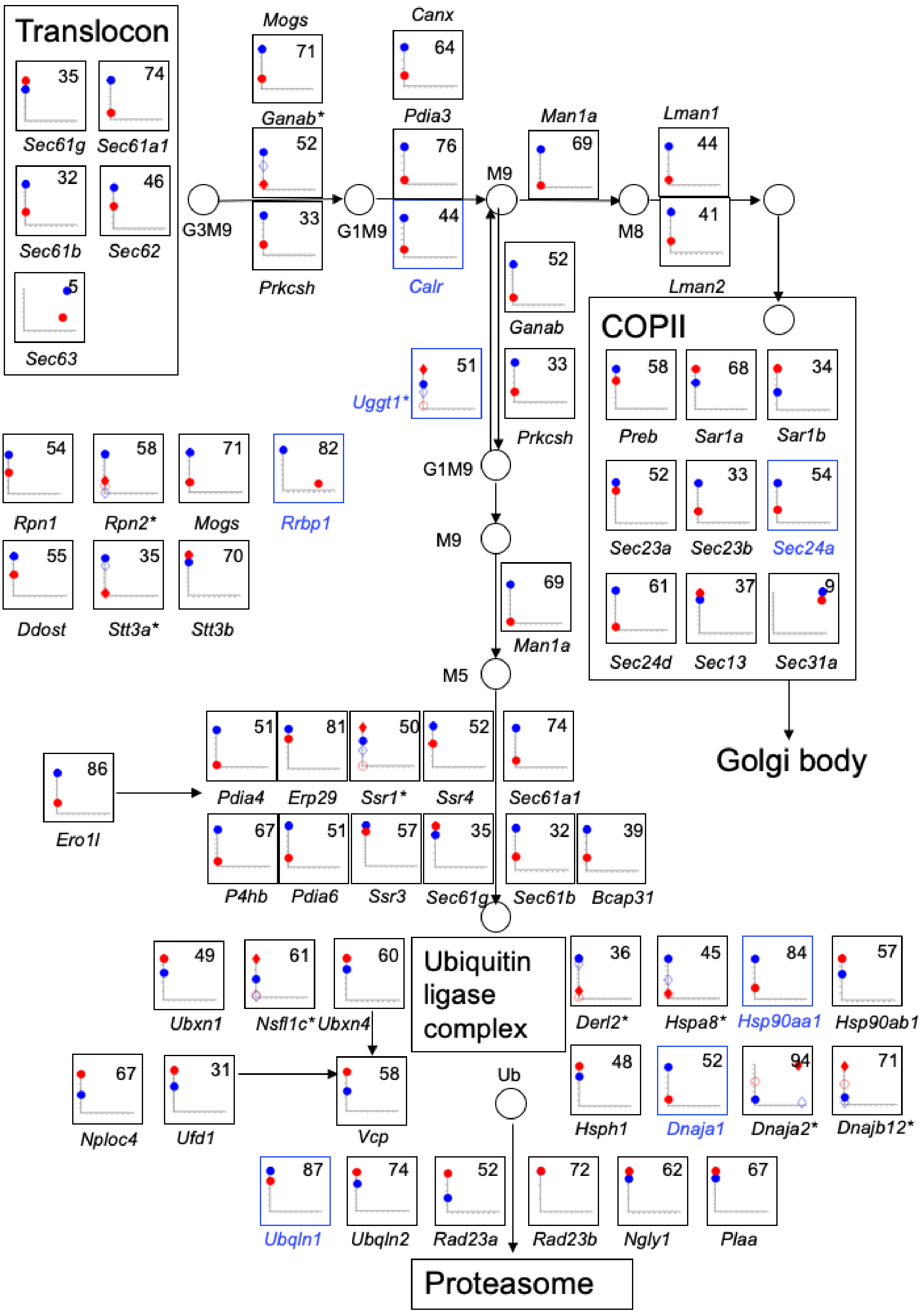

Supplementary Figure 7. Gene expression levels versus methylation ratios for each enzyme in the liver and skeletal muscle pathways. Gene expression levels are plotted against methylation ratios for genes encoding each enzyme (blue: liver-dominant, red: skeletal muscle-dominant) in the following pathways: (**a**) Glycolysis/gluconeogenesis and glycogen synthesis, (**b**) Transporters (**c**) coagulation system, (**d**) complement system (classical and lectin pathways), (**e**) urea cycle, (**f**) CYP450-related drug metabolism, (**g**) tryptophan metabolism, (**h**) primary bile acid synthesis, (**i**) alcohol degradation, (**j**) ketone body synthesis, (**k**) estrogen metabolism, (**l**) Component proteins of the sarcomere, (**m**) Enzymes of BCAA degradation, (**n**) ribosomal component proteins, (**o**) protein processing in ER. DMDEGs are colored with the gene name and the frame of the plot (blue: liver-dominant, red: skeletal muscle-dominant). Proteins indicated with * are those whose highest gene expression TSS differs between liver and skeletal muscle; the values in the two TSSs are indicated by circles and diamond-shaped dots, respectively, and the point of the TSS with the highest expression in each tissue is filled in.

Supplementary Table 1 | Relationship between TF-binding region and CpG methylation. Examine TFs with high amounts of hypomethylated CpG in the binding region. For each TF in the liver and skeletal muscle, the right-tailed Fisher's exact test was used to test whether the TF contained a lot of hypomethylated DMCpG within the binding region obtained from the ChIP-atlas. TFs that bind in the liver and significantly overlap with liver-hypomethylated DMCpGs are shown in the upper left corner of the table. TFs that bind in skeletal muscle and significantly overlap with skeletal muscle-hypomethylated DMCpGs are shown in the lower-left corner of the table. TFs that were not significant are shown in the right column. No TFs significantly overlapped with hypermethylated CpGs.

	Significantly overlapped with hypomethylated CpG	Not significant
Liver TF	Ahr, Arid1a, Arid1b, Arid2, Arntl, Bcl6, Brd4, Brd9, Cebpa, Cebpb, Clock, Cnot1, Creb1, Crebbp, Cry1, Ctcf, Ctnnb1, Cux2, Dbp, Dpf2, E2f1, E2f4, Egr1, Ep300, Esr1, Ezh2, Foxa1, Foxa2, Foxa3, Gabpa, Gata4, Gps2, Gtf2b, Gtf2e2, HBx, Hcfc1, Hdac3, Hnf1a, Hnf4a, Hnrnpu, Hsf1, Klf6, Kmt2c, Med1, Mybl1, Myc, Nanog, Ncoa2, Ncoa3, Ncor1, Ncor2, Nelfa, Nfe2l2, Nfil3, Nr0b2, Nr1d1, Nr1d2, Nr1h3, Nr1h4, Nr3c1, Nucks1, Onecut1, Pbrm1, Pknox1, Pml, Ppara, Pparg, Prox1, Rad21, Raly, Rara, Rela, Rest, Rora, Rorc, Rxra, Setdb1, Smarca4, Smc3, Srebf1, Srf, Stag1, Stag2, Stat5a, Taf3, Tbp, Tcf3, Tcf4, Tead4, Trim28, Xbp1, Yap1	Cbx5, Crtc2
Skeletal muscle TF	Arntl, Ash1l, Brd4, Cdc73, Cebpb, Chd2, Ctcf, Ctr9, Dpf3, Duxbl1, E2f4, Eed, Ep300, Esrra, Ezh2, Fosl1, Foxk1, Foxo3, Gata4, Hdac1, Hdac2, Hdac3, Hdgfrp2, Heyl, Jun, Kdm1a, Kdm6a, Kmt2a, Kmt2c, Leo1, Max, Mef2a, Mef2d, Men1, Myf5, Myod1, Myog, Nef2l2, Nr1d1, Paf1, Pax3, Pax7, Pbx1, Rbpj, Rest, Rtf1, Runx1, Sin3a, Sin3b, Six4, Smarca4, Smc3, Snai1, Sox3, Spin1, Srf, Supt16, Supt5, Supt6, Tcf12, Tcf3, Tead1, Tead4, Usf1, Wdr5, Wdr61, Yy1	-

Supplementary Table 2 | TFs of which we can compare the binding state in liver and skeletal muscle. TFs with ChIP-seq in both liver and skeletal muscle, TFs with ChIP-seq in the liver only and zero expression in the skeletal muscle, and TFs with ChIP-seq in the skeletal muscle only and zero expression in the liver are shown. For these TFs, the binding status will be compared between the liver and skeletal muscle.

Type	TF name
ChIP in Liver and Skeletal muscle	Arntl, Brd4, Cebpb, Clock, Ctcf, E2f4, Ep400, Ezh2, Foxk1, Gata4, Hdac3, Kdm1a, Kmt2c, Nfe2l2, Nr1d1, Nr3c1, Smarca4, Smc3, Srf, Tcf3, Tead4
ChIP in Liver, express only in Liver	Foxa1, Nr0b2, Onecut1
ChIP in Skeletal muscle, express only in Skeletal muscle	Fosl1, Myf5, Myod1, Myog, Pax3, Pax7

Supplementary Table 3 | Table of protein abbreviations and their names (Excel file).
